# Transcriptional control of motility enables directional movement of *Escherichia coli* in a signal gradient

**DOI:** 10.1038/s41598-017-08870-6

**Published:** 2017-08-21

**Authors:** Jayamary Divya Ravichandar, Adam G. Bower, A. Agung Julius, Cynthia H. Collins

**Affiliations:** 1 Department of Chemical and Biological Engineering, Rensselaer Polytechnic Institute, 110 8th Street, Troy, New York, 12180 United States of America; 2 Centre for Biotechnology and Interdisciplinary Studies, Rensselaer Polytechnic Institute, 110 8th Street, Troy New York, 12180 United States of America; 3 Present Address: Regeneron Pharmaceuticals, Rensselaer, New York, 12144 United States of America; 4 Department of Electrical, Computer and Systems Engineering, Rensselaer Polytechnic Institute, 110 8th Street, Troy, New York, 12180 United States of America; 5 Department of Biological Sciences, Rensselaer Polytechnic Institute, 110 8th Street, Troy, New York, 12180 United States of America

## Abstract

Manipulation of cellular motility using a target signal can facilitate the development of biosensors or microbe-powered biorobots. Here, we engineered signal-dependent motility in *Escherichia coli* via the transcriptional control of a key motility gene. Without manipulating chemotaxis, signal-dependent switching of motility, either on or off, led to population-level directional movement of cells up or down a signal gradient. We developed a mathematical model that captures the behaviour of the cells, enables identification of key parameters controlling system behaviour, and facilitates predictive-design of motility-based pattern formation. We demonstrated that motility of the receiver strains could be controlled by a sender strain generating a signal gradient. The modular quorum sensing-dependent architecture for interfacing different senders with receivers enabled a broad range of systems-level behaviours. The directional control of motility, especially combined with the potential to incorporate tuneable sensors and more complex sensing-logic, may lead to tools for novel biosensing and targeted-delivery applications.

## Introduction

Cellular motility is a key microbial behaviour with a broad range of functions in natural systems, including navigation of the environment^1^, biofilm formation^2^, and control of biodiversity in consortia^3^. Bacteria move in a self-propelled manner by drawing energy from their surroundings and have developed mechanisms to effectively navigate their environments. Bacteria also monitor their environment and respond to changes therin^4, 5^. Controlling cellular motility in response to an external signal can facilitate the development of biosensors^6–8^ or micromachines that use microbes to enable movement in microfluidic environments^9, 10^, with potential applications as targeted-delivery agents.

*Escherichia coli* swim through their environment powered by the rotation of their flagella^11^. The flagella are self-assembled structures made up of a hook, filament and motor^12^. The hook is flexible while the filament is rigid and its shape is determined by the direction of flagellar rotation. The motor is powered by a proton gradient that generates the torque required for flagellar rotation^13, 14^. In the absence of attractants or repellents to guide the direction of movement, bacteria follow a random walk pattern involving a series of runs and tumbles determined by the direction of flagellar rotation^1^. Chemoreceptors bind to attractants resulting in a change in the phosphorylation state of proteins that control the direction of flagellar rotation^15, 16^, reducing the tumbling frequency of cells, and allowing cells to run in more direct paths towards attractants^17, 18^.

Different strategies have been pursued to engineer motility in *E*. *coli* in response to target signal molecules^19^. Several efforts have used *E*. *coli* strains rendered non-motile via deletion of motility proteins and then restored motility via inducible expression of the deleted gene from a plasmid^20, 21^. For example, control of motility was achieved in a *cheZ*-deletion *E*. *coli* strain by using a theophylline-sensitive riboswitch to control expression of CheZ, a protein that controls cellular tumbling rate^22^. Control of directional motility in response to target compounds has been achieved by engineering *E*. *coli* chemoreceptors to recognize target compounds via directed evolution^23^, rational design of the chemoreceptor specificity^24^, and designing hybrid chemoreceptors consisting of an *E*. *coli* signalling domain and a sensory domain from other species that recognizes a target compound^25^. While such strategies for controlling directional movement targeting *E*. *coli*’*s* chemotactic network have led to some success, the limited number of natural chemoreceptor scaffolds imposes constraints on ligands that can be targeted. Such engineering challenges have led to alternative approaches, such as converting the desired target to a compound recognized by *E*. *coli*’*s* native chemotactic machinery^26^.

Engineering directional motility in response to signal molecules non-native to *E*. *coli*’s sensing machinery, without manipulation of chemotaxis, would expand the use of bacteria in sensing and actuation applications. Interestingly, some enzymes have been observed to exhibit an increase in diffusivity that correlates to increasing concentrations of their substrate. The substrate concentration-dependent enhancement in diffusivity enables directional movement of the enzymes up gradients of their cognate signals^26^. For example, urease exhibits an increase in diffusivity with increasing concentrations of its substrate urea, and this enables directional movement of urease up a substrate gradient^27, 28^. Similar directional migration was observed with catalase molecules in a hydrogen peroxide gradient^28^. This ability of enzymes to enable directed self-propulsion has been harnessed to drive polystyrene beads coated with urease or catalase up the gradients of their cognate substrates^29^. This mechanism of directional movement resulting from substrate concentration-dependent enhanced diffusivity could be applied to engineer directional movement of cells in a signal gradient by enhancing cellular diffusivity in the presence of a signal. We hypothesized that transcriptional control of a key motility gene in response to a signal would allow signal-dependent manipulation of cellular diffusivity and enable population-level directional movement of cells in a signal gradient.

Natural quorum sensing (QS) systems enable cell density-dependent control of gene expression in bacteria based on the production and detection of QS signal molecules^30, 31^. QS systems have been used by synthetic biologists for tuneable transcriptional control of gene expression^32, 33^, and the expression of a QS-signal synthase in *E*. *coli* has been shown to generate a signal gradient across a petri dish^34^. QS systems have been widely used for construction of genetic circuits in individual cells^35, 36^ and to enable communication in synthetic consortia^37, 38^. Previous work has used QS regulatory elements to control motility in *E*. *coli* strains lacking *cheZ* ^20^ or *motB* ^21^, where the missing motility gene was expressed from a QS-signal inducible promoter. The ability to reliably control gene expression and manipulate cells in cell-generated gradients make QS regulatory components ideal tools for examining transcriptional control of motility in *E*. *coli* in a signal gradient.

Here, we engineered *E. coli* strains where motility is tightly regulated by transcriptional control of the motor protein, MotA, and is induced by a QS signal molecule. We demonstrate robust directional control of motility in the engineered ‘receiver’ cells that was not only achieved in a gradient of exogenously added signal but also in a bio-generated gradient of the signal produced by ‘sender’ cells. We show that our sender-receiver architecture is modular and can be used to generate a range of sensitivity and responses to the signal. Further, we describe a mathematical model that provides insight into key aspects of system behaviour and enables predictive-design of motility-based pattern formation by cells.

## Results

### Design and characterization of signal-molecule dependent motility in *E*. *coli*

**Figure 1:**
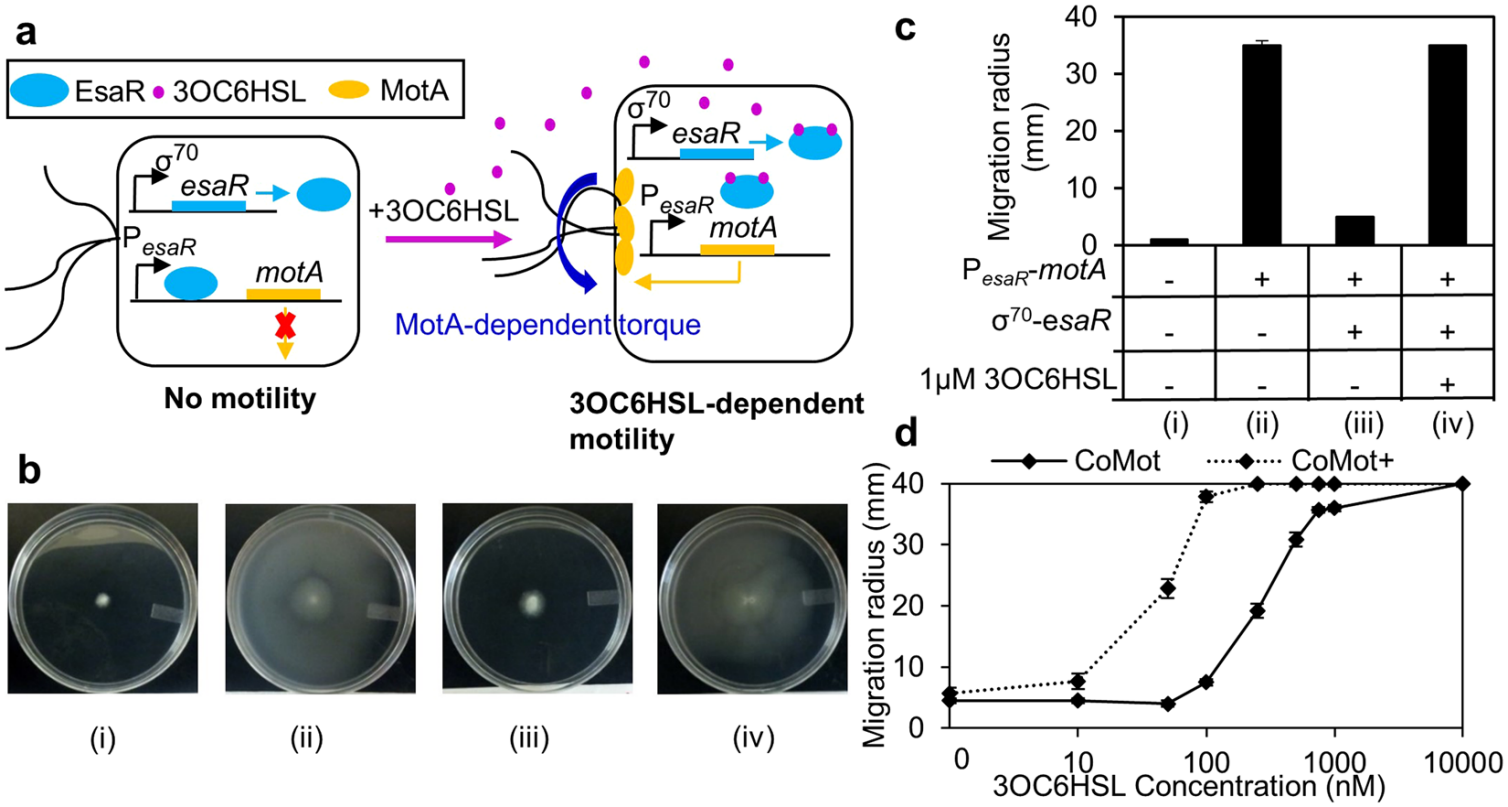
Engineered CoMot strains display 3OC6HSL-dependent motility: (**a**) Illustration of 3OC6HSL-dependent *motA* expression in the CoMot strain. Expression of *motA* is under the control of the P_*esaR*_ promoter. *esaR* is constitutively expressed from a σ^70^-dependent promoter and represses P_*esaR*_. In the absence of 3OC6HSL, *motA* expression is repressed and the cell is non-motile. Following addition of 3OC6HSL, P_*esaR*_ is de-repressed and *motA* is expressed. MotA generates the torque required to rotate the flagella and cellular motility is restored. (**b**) Plates were inoculated with (i) Δ*motA*, (ii) Δ*motA* transformed with plasmids containing P_*esaR*_-*motA* (iii) CoMot cells (Δ*motA* transformed with plasmids containing P_*esaR*_-*motA* and P_σ70_-*esaR*) on plates without 3OC6HSL and (iv) CoMot cells on plates with 1 μM 3OC6HSL and incubated at 30 °C for 36 h. Representative plate images are shown. (**c**) Migration radius was measured as the distance between the inoculation point and the visible edge of migration of cells on the plate for cases (i–iv). Error bars represent one standard deviation from the mean migration radius of three biological replicates. (**d**) Plates with 3OC6HSL concentration ranging from 0 to 10 μM were inoculated with CoMot or CoMot+ (Δ*motA* transformed with plasmids containing P_*esaR*_-*motA* and P_σ70_-*esaR-D9*1*G*). The migration radius was measured after 36 h at 30 °C. Error bars represent one standard deviation from the mean migration radius of three biological replicates.

To build a system where the motility of *E*. *coli* is transcriptionally regulated by QS components, we used the *esa* QS system to control expression of MotA in an *E*. *coli motA* deletion strain (∆*motA*)^39^. MotA is a motor protein that provides a channel for the proton gradient required for generation of torque^40^. ∆*motA* strains can build flagella but are non-motile because they are unable to generate the torque required for flagellar rotation^14^. Expression of *motA* from a plasmid has been shown to restore motility in ∆*motA* strains^41^. Previous efforts to regulate motility using the activation-based *lux* QS system faced challenges in achieving tight regulation of the target gene and basal expression of the gene was sufficient to restore motility in the absence of the signal^21^. The *esa* QS system is from the plant pathogen *Pantoea stewartii* ^42^, and has been shown to provide tight regulation of genes downstream of the esaR promoter (P_*esaR*_)^43^. The QS regulator EsaR represses P_esaR_ expression by binding to the promoter. The addition of acyl-homoserine lactone QS signal molecule, 3-oxo-hexanoyl homoserine lactone (3OC6HSL)^43^, induces gene expression from P_*esaR*_ by triggering EsaR to release the promoter and allow RNA polymerase to bind and initiate transcription. Here, we constructed a two-plasmid system in ∆*motA* cells consisting of an EsaR-expression plasmid and a plasmid in which *motA* is placed under the control of P_*esaR*_. As shown in Fig. 1a, expression of *motA* is repressed by the transcriptional repressor (EsaR) in the absence of 3OC6HSL. In the presence of 3OC6HSL, EsaR is expected to dissociate from the promoter triggering expression of *motA*, thereby restoring motility in ∆*motA* cells. The green fluorescent protein was also placed downstream of P_*esaR*_ to allow characterization of expression from P_*esaR*_, if *motA* expression was not sufficient to provide detectable motility in our assays. This strain is designated as the **Co**mmunication-dependent **Mot**ility (CoMot) strain.

A motility assay using semi-solid agar plates that allow cells, which were inoculated by stabbing 1 μL of cells into the agar, to migrate through the media, was used to quantify motility. The migration radius was measured as the distance between the inoculation point and the visible edge of the migrating cells on the plate. As expected, migration was not observed on plates inoculated with ∆*motA* cells (Fig. 1b and c; case i). A migration radius of 35 mm was observed on plates inoculated with ∆*motA* cells transformed with a plasmid containing P_*esaR*_ *-motA* (case ii), indicating that constitutive expression of MotA from P_*esaR*_ was sufficient to restore motility in ∆*motA* cells. To assess if 3OC6HSL-inducible motility could be achieved in CoMot cells, they were inoculated on plates with and without 3OC6HSL. As seen in case iii, a migration radius of only 5 mm was observed in the absence of 3OC6HSL. This was comparable to the migration radius of ∆*motA* cells, demonstrating that motility in the absence of 3OC6HSL is minimal. A seven-fold increase in the migration radius exhibited by CoMot cells was observed in the presence of micromolar concentrations of 3OC6HSL (case iv), indicating that engineered cells exhibit signal-molecule dependent motility.

CoMot cells were inoculated on plates with 0, 10, 50, 100, 250, 500, 750, 1000 and 10000 nM 3OC6HSL, to assess their sensitivity to the signal molecule. As shown in Fig. 1d, an increase in migration radius was observed with increasing 3OC6HSL concentrations, where 250 nM 3OC6HSL was required to observe a migration radius larger than the background migration radius observed in the absence of 3OC6HSL (*p* = 0.0017). To increase the 3OC6HSL sensitivity of the cells, we replaced the transcriptional repressor EsaR with a variant, EsaR-D91G and designated this strain as CoMot+. *E*. *coli* cells with EsaR-D91G have been reported to display a 100-fold higher sensitivity to 3OC6HSL compared to wild-type EsaR in a luminescence-based promoter assay^43^. As seen in Fig. 1d, CoMot+ cells required 50 nM 3OC6HSL to display a migration radius above background (*p* = 0.0028), demonstrating that the CoMot+ strain does exhibit increased sensitivity to 3OC6HSL. In addition, 10000 nM of 3OC6HSL was required for CoMot cells to reach the edge of the plate in 36 hours, while only 250 nM was required for CoMot+ cells (Fig. 1d).

### Characterization of directional movement of the CoMot variants in a 3OC6HSL gradient

**Figure 2:**
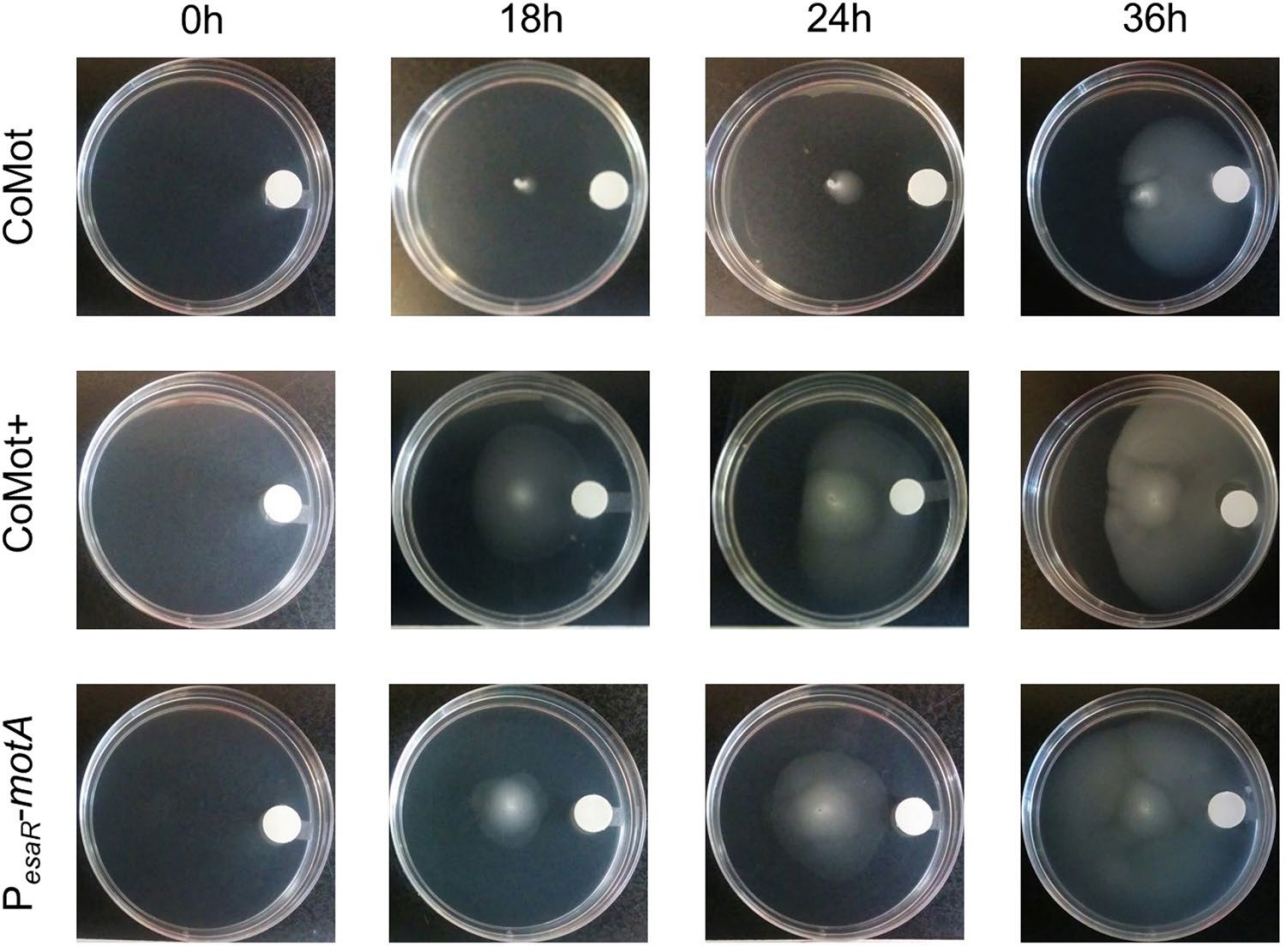
CoMot and CoMot+ cells in a 3OC6HSL gradient show directional movement towards the 3OC6HSL source: A 3OC6HSL gradient was established by adding 0.02 μmoles of 3OC6HSL to a Whatmann membrane and allowing it to diffuse for 8 h. 1 μM of 3OC6HSL would be the final concentration if 0.02 μmoles of 3OC6HSL diffused uniformly through the plate. CoMot, CoMot+ or cells that constitutively express *motA* (∆*motA* transformed with a plasmid containing P_*esaR*_-*motA*) were then inoculated at the centre of the plate. Images were obtained following 0, 18, 24 and 36 h of incubation at 30 °C. The assay was run in triplicate for each strain and representative images are shown.

To assess if CoMot and CoMot+ cells display directional movement in a signal gradient, 0.02 μmoles of 3OC6HSL was added on a membrane (3OC6HSL source), placed 1.25 cm from the edge of the plate, and allowed to diffuse and establish a gradient for 8 h prior to inoculation of cells at the centre of the plate. 1 μM of 3OC6HSL would be the final concentration if the 0.02 μmoles diffused uniformly through the 25 mL plate. As shown in Fig. 2, both CoMot variants reached the edge of the plate (40 mm) in the forward direction towards the 3OC6HSL source by 36 h. Here, we define forward migration distance as distance between the inoculation point and the visible edge of cells that have migrated up the signal gradient. The reverse migration distance (distance between the inoculation point and the visible edge of cells that have migrated down the signal gradient) was 10 mm for CoMot and 18 mm for CoMot+. Therefore, directional movement of both CoMot variants up the 3OC6HSL gradient was observed. Directional movement was not displayed by ∆*motA* cells that contain P_*esaR*_-*motA* and constitutively express *motA* (Fig. 2), indicating that regulation by EsaR or EsaR-D91G in the 3OC6HSL gradient is required for directional movement.

**Figure 3:**
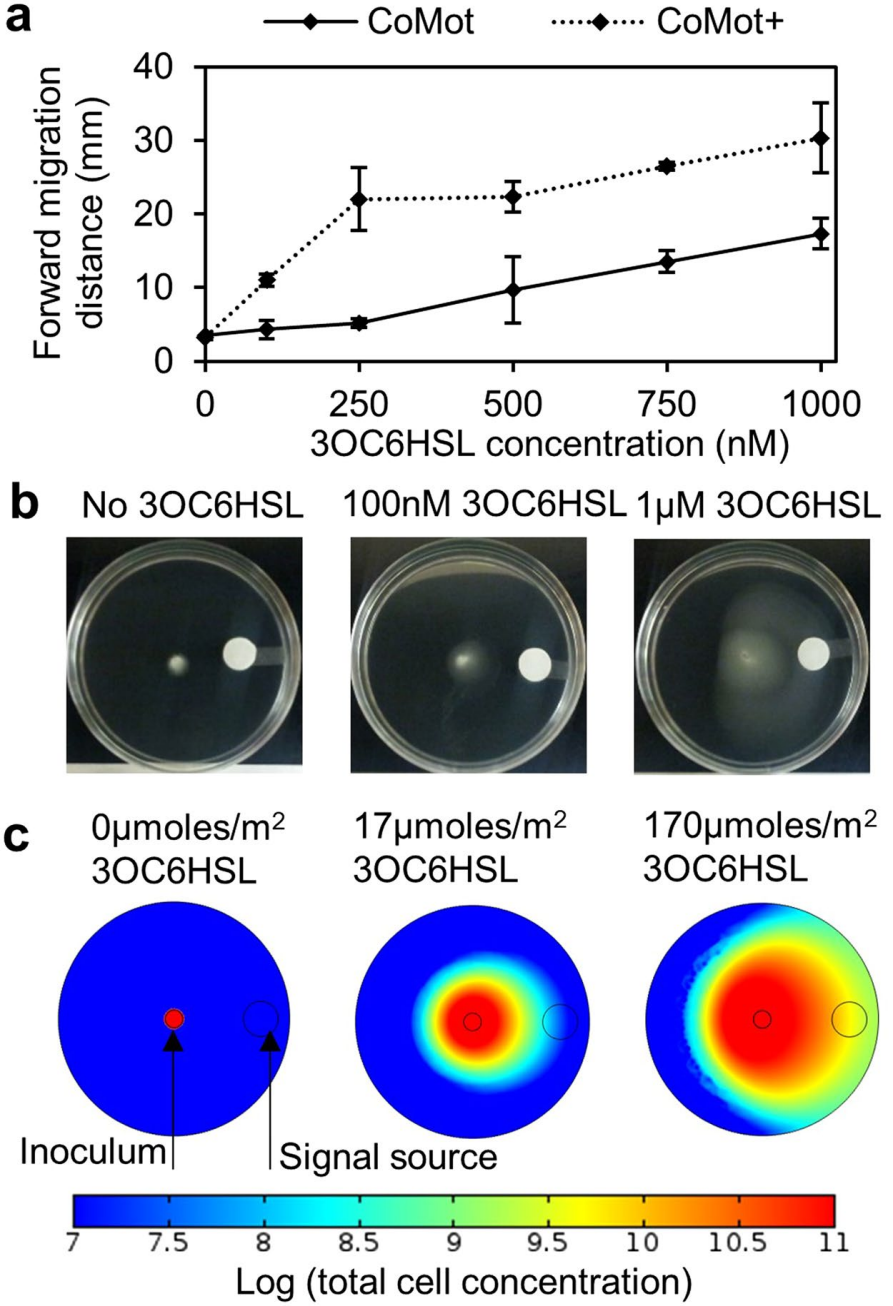
Motility assays and simulations show 3OC6HSL-dependent directional movement of cells in a 3OC6HSL gradient: (**a**) 3OC6HSL gradients were established by adding 0–0.02 μmoles of 3OC6HSL on a Whatmann membrane and allowing it to diffuse for 8 h. CoMot and CoMot+ cells were then inoculated at the centre of the plate. The forward migration distance was measured as the distance between the inoculation point and the visible edge of migration of cells up the signal gradient after 24 h of incubation at 30 °C. Error bars represent one standard deviation from the mean forward migration distance of three biological replicates. (**b**) Representative images of plates inoculated with CoMot+ cells. (**c**) Results from simulation of the migration response of cells in a similar set up as in (b). Signal gradients were simulated by using 0, 17 or 170 μmoles/m^2^ of the signal near the edge of the plate. 3.5*10^7^ CoMot+ cells/m^2^ was used as the inoculum at the centre of the plate. The log_10_ of the total cell concentration after a simulation time of 24 h is shown in the images.

We then examined the sensitivity of CoMot and CoMot+ cells in gradients established using varying amounts of 3OC6HSL. As seen in Fig. 3a, both CoMot variants showed an increase in the forward migration distance with increasing 3OC6HSL concentrations. Similar to the uniform 3OC6HSL-titration results, a lower 3OC6HSL concentration was required to observe forward migration distances greater than background levels with the CoMot+ (100 nM) than CoMot cells (500 nM). Both strains displayed directional movement towards the 3OC6HSL source (Fig. 3b). We also observed that the forward migration distance decreased when cells were inoculated at increasing distances from the 3OC6HSL source indicating that motility response is affected by the spatial arrangement of the signal and cells (Supplementary Fig. S1).

### Modelling of signal molecule-guided bacterial motility

To gain insight into the key parameters controlling motility in the engineered strains and to identify factors contributing to the observed directional movement, we developed a mathematical model. The distribution of CoMot cells in response to the signal molecule was modelled using Equations (1–3). We used a Michaelis Menten-type term (*term III*) to model the rate of switching from static (*s*) to motile cells (*m*) in the presence of the signal molecule (*A*) and an inhibition-kinetics equation to capture the switching from motile to static cells (*term IV*). Parameters *k*_1_ and *γ* capture the maximum rate of switching from static to motile and motile to static, respectively. *K*_2_ and *K*_4_ define the sensitivity of cells to *A*. The displacement of motile cells is modelled via a diffusion term (*term I*), where *D*_*m*_ is the effective diffusivity of cells. We used Monod kinetics to capture the exponential growth of cells. λ represents growth rate in *term II*. Diffusion of the signal molecule is captured in term V, where *D*_*a*_ represents the diffusivity of *A*. A two-dimensional version of the experiment was modelled using geometry (plate size and location of signal and cells) similar to the experimental setup. The model was simulated using parameters estimated experimentally or from the literature (Supplementary Table S1). When experimental quantification was not possible and when quantitative values were unavailable in literature parameter values were chosen based on educated guesses in biologically feasible regimes. These values were then tuned to fit experimental findings when required.

1 $$\frac{\partial m}{\partial t}=\,\mathop{\overbrace{{D}_{m}(\frac{{\partial }^{2}m}{\partial {x}^{2}}+\frac{{\partial }^{2}m}{\partial {y}^{2}})}}\limits^{I}+\mathop{\overbrace{\lambda m}}\limits^{II}\,+\mathop{\overbrace{(\frac{{k}_{1}A\,}{{K}_{2}+A})s}}\limits^{III}-\mathop{\overbrace{(\frac{\gamma }{1+\frac{A}{{K}_{4}}})m}}\limits^{IV}$$

2 $$\frac{\partial s}{\partial t}=\mathop{\overbrace{\lambda s}}\limits^{II}-\mathop{\overbrace{(\frac{{k}_{1}A\,}{{K}_{2}+A})s}}\limits^{III}+\mathop{\overbrace{(\frac{\gamma }{1+\frac{A}{{K}_{4}}})m}}\limits^{IV}$$

3 $$\frac{\partial A}{\partial t}=\mathop{\overbrace{{D}_{a}(\frac{{\partial }^{2}A}{\partial {x}^{2}}+\frac{{\partial }^{2}A}{\partial {y}^{2}})}}\limits^{V}$$

We started by simulating the migration response of CoMot+ cells in gradients established using 0, 17 or 170 µmole/m^2^ of the signal molecule. In the motility assays, 0.02 μmoles of 3OC6HSL were added to the membrane and used to generate a gradient equivalent to 1 μM. In the 2D simulations, 0.02 μmoles was initially distributed across the area of the membrane (1.13*10^−4^ m^2^). Therefore, an initial signal concentration of 170 μmoles/m^2^ (0.02 μmoles/1.13*10^−4^ m^2^) on the membrane was used, where 3.8 μmoles/m^2^ would be the final concentration if the signal diffused uniformly across the simulated area of the plate. Images after a simulated time of 24 h are shown in Fig. 3c. In the absence of the signal, simulated cells remained at the inoculation point. Similar to experimental observations (Fig. 3b), an increase in movement of simulated cells towards the signal source was observed with increase in signal concentration. To model the difference in 3OC6HSL sensitivity of CoMot and CoMot+ cells, we increased the sensitivity parameter, *K*_2_ from 1 to 100 nmole/cm^2^. Time course simulations of CoMot and CoMot+ cells in a gradient established using 170 µmole/m^2^ of the signal are shown in Supplementary Fig. S2. CoMot+ cells displayed approximately 2-fold higher forward migration distance than CoMot cells after a simulation time of 24 h, where a cell concentration ≥10^8^ was used as the cut off for the migration distance in the simulations. Thus, our model is representative of the system and captures key system properties - signal-molecule dependent directional movement in a gradient and the difference in the 3OC6HSL sensitivity between CoMot and CoMot+.

**Figure 4:**
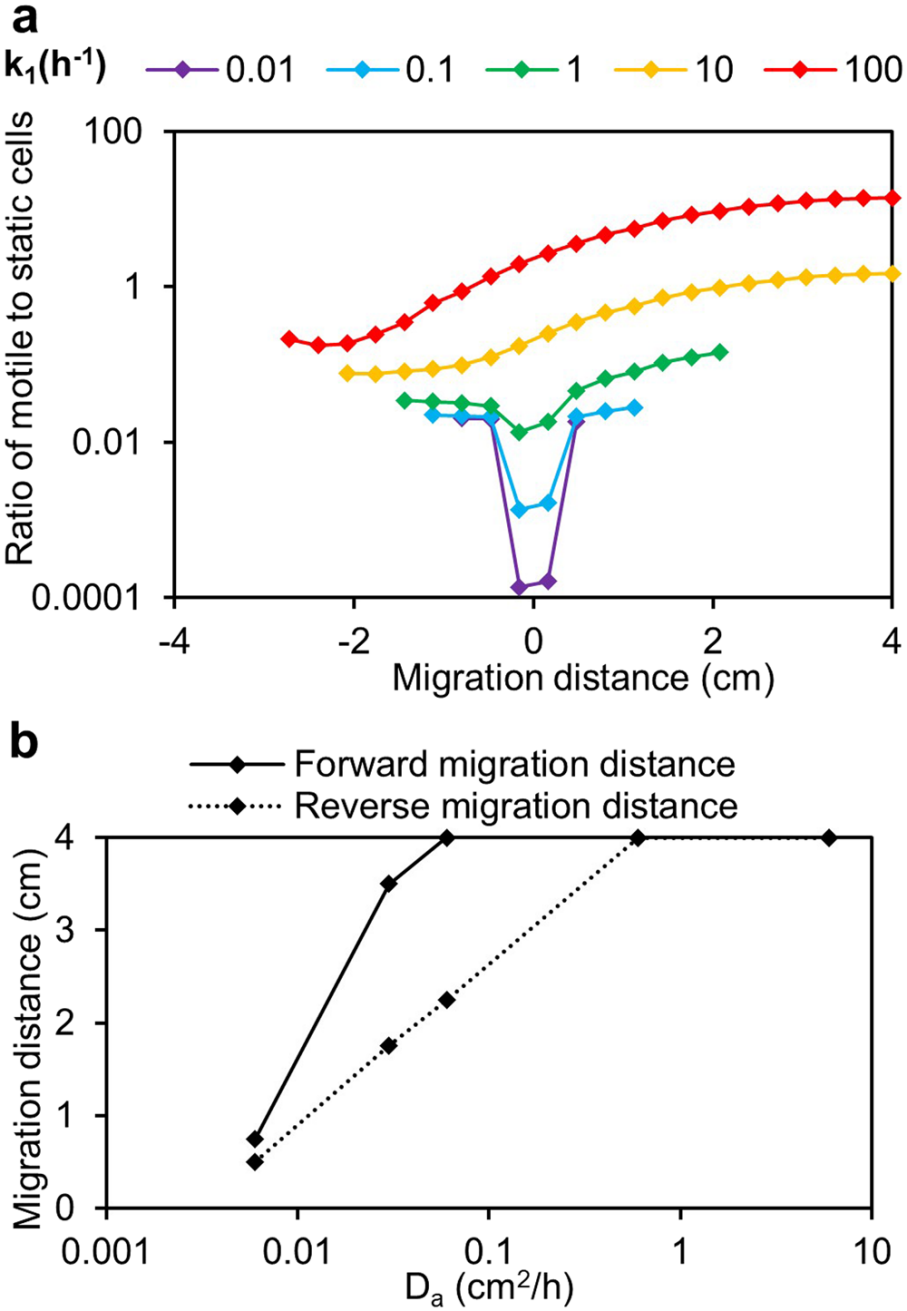
The rate of switching from static to motile (*k*_1_) and the diffusivity of the signal (*D*_*a*_) are keys parameters controlling migration of cells: In the simulations, the gradient was established using 85 μmoles/m^2^ of the signal near edge of the plate (migration distance = 4 cm). 3.5*10^7^ static cells/m^2^ was used as the inoculum at the centre of the plate (migration distance = 0 cm). (**a**) *k*_1_ was varied from 0.01–100 h^−1^ while all other parameters were held constant at values defined in the base parameter set. For each value of *k*_*1*_, the ratio of motile to static cells (m/s) across the diameter of the plate (migration distance = −4 to 4 cm) was plotted after a simulation time of 24 h. The ratio was only calculated at points with a total cell concentration ≥10^8^ cells/m^2^. (**b**) Simulations were run varying D_a_ from 0.01−10 cm^2^/h while holding all other parameters constant. Forward and reverse migration distances were measured as distances from the inoculation point (migration distance = 0) towards and away from the signal source at which a total cell concentration ≥10^8^ cells/m^2^ was observed after a simulation time of 24 h.

To understand the effect of the two switching rates on system behaviour, we varied *k*_1_ and *γ* and plotted the ratio of motile to static cells (Fig. 4a and supplementary Fig. S3a). An increase in the motile to static cell ratio was observed with increasing *k*_1_ and decreasing *γ*. The increase in this ratio leads to an increase in both the forward and reverse migration distances. Thus, varying the switching rates allow for tuning of the magnitude of motility response of the cells. Our simulations showed that m/s increases as cells move towards the 3OC6HSL source, indicating that motile cells dominate the population up the signal gradient and static cells dominate down the gradient. Thus, a cell once motile, though capable of moving in any random direction, remains motile if it happens to move up the gradient, but switches to static if it migrates down the gradient and into a region of low signal. This static population continues to accumulate and grow. Directional movement then results from population-level movement of motile cells towards the signal source.

Simulations varying the signal diffusivity (*D*_*a*_) were run to assess its effect on the established gradient on migration response. The signal concentration across the plate (Supplementary Fig. S3b) and the forward and reverse migration distances were examined (Fig. 4b) for each simulated *D*_*a*_. Directional movement, as indicated by a greater forward compared to reverse migration distance, was only observed with *D*_*a*_ sufficient to establish a gradient (*D*_*a*_ < 0.6 cm^2^/h). It is thus evident that the established signal gradient drives the directional movement of the cells.

*K*_2_ and *K*_4_ capture the 3OC6HSL-sensitivity of cells when switching from static to motile and motile to static, respectively. Our simulations showed that a 10^4^-fold increase in *K*_2_ resulted in a 1000-fold increase in the signal concentration required to achieve a forward migration distance equivalent to reaching the edge of the plate in 24 hours, while a 10^8^-fold increase in *K*_4_ resulted in only a 50-fold increase (Supplementary Fig. S3c,d). Thus, the sensitivity of the cells to 3OC6HSL when switching from static to motile has a larger effect on overall system behaviour than the sensitivity when switching from motile to static. At high *K*_4_ (*K*_4_ > 2500 nmole/cm^2^), where switching from motile to static becomes independent of 3OC6HSL, directional movement of cells was still observed. Here, dilution of MotA as cells grow and divide leads to switching from motile to static if the local concentration of 3OC6HSL is not sufficient to induce additional *motA* expression. However, in simulations with low *K*_4_ (*K*_4_ < 0.0025 nmole/cm^2^), directional behaviour was not observed (Supplementary Fig. 3e,f). In this case, cells never switch back to static once they become motile and thus continue migrating across the plate. Overall, these simulations indicate that while it is essential that cells are able to switch from motile to static (*term IV*), observed experimental behaviour and directional movement can be captured if *term IV* is modelled as dependent or independent of signal concentration.

We observed that both the forward and reverse migration distances increased when the effective diffusivity of cells (*D*_*m*_) was increased (Supplementary Fig. S3g). Directional movement towards the signal was not observed at values of *D*_*m*_ 10-fold greater than the experimentally estimated 0.1 cm^2^/h. This could be because in the simulated geometry-scale at high *D*_*m*_, once motile, the diffusivity of cells was sufficient to keep them motile regardless of the local signal concentration. These studies have shown that the distribution of cells depends both on the parameters regulating motility and the established gradient.

### Model-guided pattern formation

**Figure 5:**
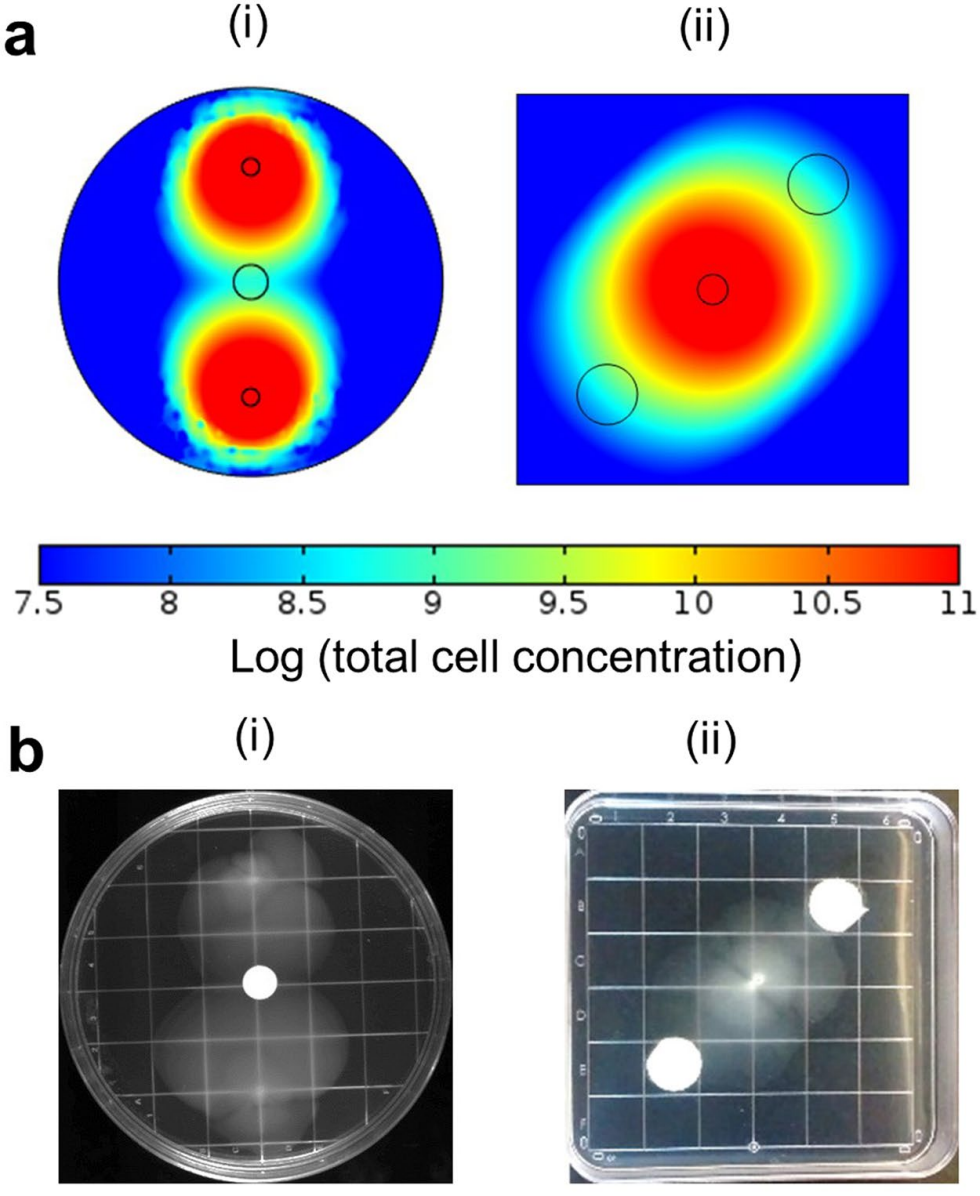
Predictive design and experimental verification of motility-induced pattern formation: (**a**) Results from simulation of the patterns formed by cells in response to different spatial arrangements of signals and starting inoculum of cells. (i) 3.5*10^7^ CoMot+ cells/m^2^ were used as the inoculum at opposite edges of a circular plate and a gradient was established using 85 μmoles/m^2^ of the signal at the centre of the plate. 85 μmoles/m^2^ of the signal is equivalent to an experimental 3OC6HSL concentration of 500 nM. Images were obtained after a simulation time of 36 h. (ii) 3.5*10^7^ CoMot+ cells/m^2^ were used as the inoculum at the centre of a square plate in which the signal gradient was established using two signal sources along a diagonal. 85 μmoles/m^2^ of the signal was used for each source to establish the gradient. Images were obtained after a simulation time of 36 h. (**b**) Experimental verification of patterns predicted by the model. (i) The equivalent of 500 nM of 3OC6HSL was added on a membrane 12 h prior to inoculation of CoMot+ cells at opposite edges of the plate. (ii) Membranes with the equivalent of 500 nM of 3OC6HSL were placed along the diagonal of the plate 12 h prior to inoculation of CoMot+ cells at the centre. Each pattern was experimentally verified in triplicate and representative images obtained after 36 h of incubation at 30 °C are shown.

We next explored if our model can be used to predict cell distribution patterns formed in response to changing the spatial arrangement of the signal and cells. Representative patterns of cell distribution that were simulated under different spatial arrangements of signal and cells and tested in similar experimental set ups are shown in Fig. 5. In case (i), the pattern predicted to be formed by cells inoculated diametrically opposite to each other in a plate with a signal source at its centre is in agreement with the pattern experimentally observed on a petri dish with CoMot+ cells inoculated in a similar set up. In case (ii), we were able to successfully predict the pattern formed by cells inoculated at the centre of a square petri dish with signal sources along a diagonal. We were thus able to predict the distribution of cells in response to different initial arrangements of the signal and cells, thereby enabling predictive-design of pattern formation by cells.

### Engineering CoMot variants to migrate down a 3OC6HSL gradient

**Figure 6:**
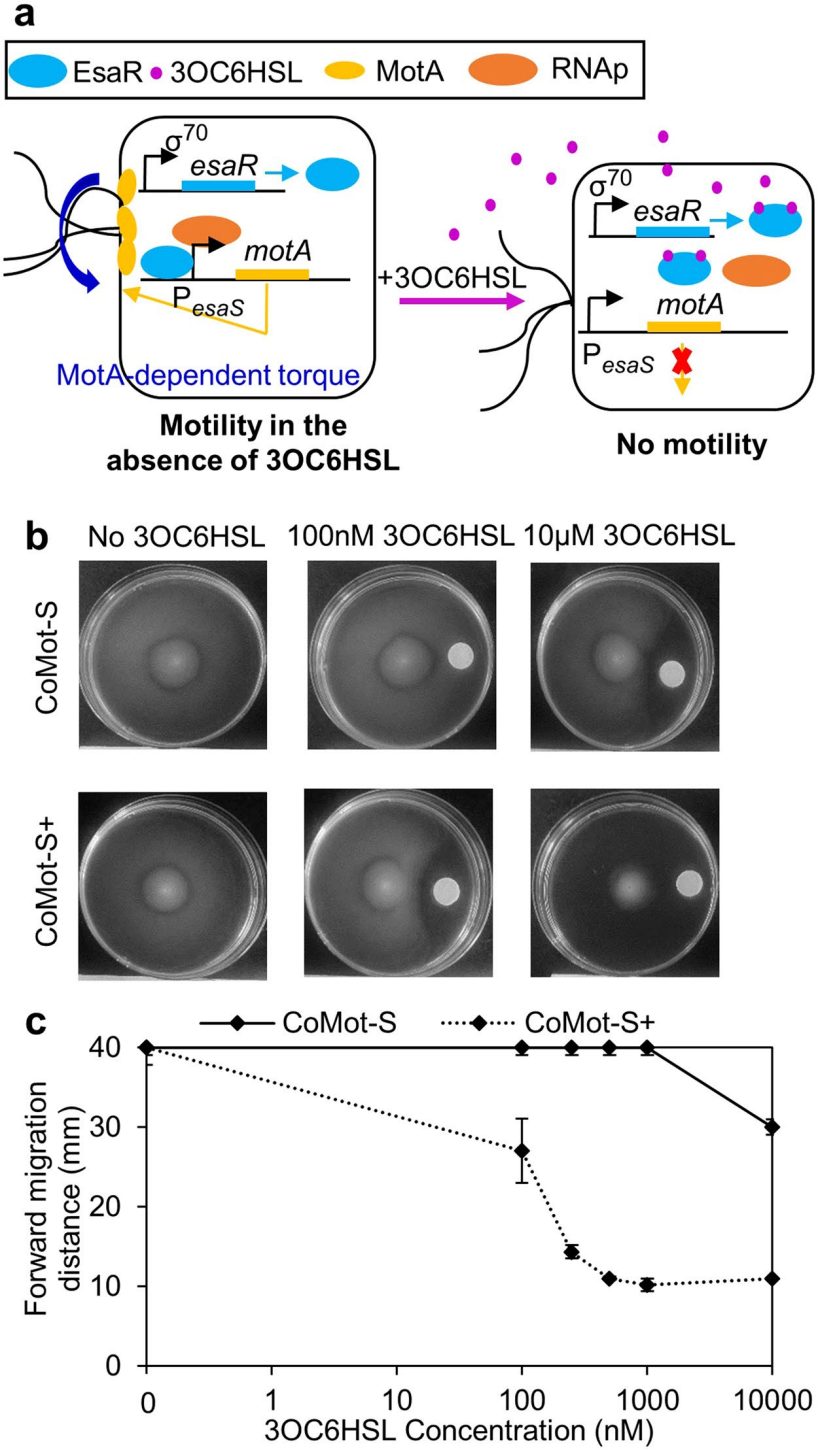
Characterization of the migration response of CoMot-S and CoMot-S+ cells in a 3OC6HSL gradient shows directional movement of cells away from a 3OC6HSL source: (**a**) Illustration of 3OC6HSL-dependent *motA* expression in the CoMot-S strain. Expression of *motA* is under the control of the P_*esaS*_ promoter. *esaR* is constitutively expressed from a σ^70^-dependent promoter and activates expression from P_*esaS*_. In the absence of 3OC6HSL, *motA* is expressed and motility is restored in the Δ*motA* cells. Following addition of 3OC6HSL, EsaR unbinds from P_*esaS*_. *motA* expression decreases and the cells become non-motile. (**b**) 3OC6HSL gradients were established by adding 0–53 μg of 3OC6HSL on a Whatmann membrane and allowing it to diffuse for 8 h. 10 μM of 3OC6HSL would be the final concentration if 53 µg of 3OC6HSL diffused uniformly through the plate. CoMot-S (Δ*motA* transformed with plasmids containing P_*esaS*_-*motA* and P_σ70_-*esaR*) or CoMot-S+ (Δ*motA* transformed with plasmids containing P_*esaS*_-*motA* and P_σ70_-*esaR-D91G*) cells were then inoculated at the centre of the plate. Representative plate images obtained after 24 h of incubation at 30 °C. (**c**) Forward migration distance was measured as the distance between the inoculation point and the visible edge of migration of cells up the signal gradient after 24 h of incubation at 30 °C. Error bars represent one standard deviation from the mean forward migration distance of three biological replicates.

We sought to engineer a strain that migrates down a 3OC6HSL gradient. The P_*esaS*_ promoter, which is activated by EsaR in the absence of 3OC6HSL^44^, was used to control expression of MotA. We predicted that this new strain, CoMot-S, would express MotA and be motile in the absence of 3OC6HSL and that motility would decrease with increasing 3OC6HSL due to dissociation of 3OC6HSL-bound EsaR from P_*esaS*_ (Fig. 6a). CoMot-S and CoMot-S+ cells with wild-type EsaR and EsaR-D91G, respectively, were inoculated on plates with uniform 3OC6HSL concentrations ranging from 0–10 µM (Supplementary Fig. S4). In the absence of 3OC6HSL, migration was observed for both strains. Approximately 1 µM of 3OC6HSL was required to observe a migration radius significantly smaller (CoMot *p* = 0.02, CoMot+ *p* = 0.016) than that observed in the absence of 3OC6HSL after 24 h of incubation at 30 °C. However, no difference in the migration radius of CoMot-S cells was observed in the presence or absence of 3OC6HSL after 36 h of incubation. The migration radius of CoMot-S+ cells, on the other hand, decreased 2.7-fold in the presence of 10 μM 3OC6HSL. Leaky expression of *motA* from the activation-based P_*esaS*_-controlled system was likely sufficient to restore motility in the CoMot-S strains even in the presence of 3OC6HSL. Despite the incomplete control of motility, we decided to assess whether we would observe directional motility of CoMot-S and CoMot-S+ down a 3OC6HSL gradient. We inoculated CoMot-S strains on plates with gradients established using 3OC6HSL concentrations equivalent to 0, 100, 250, 500, 1,000 and 10,000 nM. As seen in Fig. 6b, in the absence of 3OC6HSL, the forward and reverse migration distances are similar for the CoMot-S strains. In the presence of 3OC6HSL, migration to the edge of the plate away from the source (reverse migration distance) was observed under all conditions except for CoMot-S+ at 10 μM 3OC6HSL. A decrease in the forward migration distance with increasing 3OC6HSL concentration was observed with CoMot-S+, while 10 µM 3OC6HSL was required to see a decrease in forward migration distance with CoMot-S cells after 24 h of incubation (Fig. 6c). The directional movement away from the source was observed even after 36 h of incubation despite the previously observed migration of the CoMot-S strains in the uniform 3OC6HSL motility assays. The CoMot-S strains are capable of migrating down a 3OC6HSL gradient, thereby adding to our repertoire of ways to control the directional motility of *E*. *coli*.

### Design and characterization of a sender-receiver system

To test if motility in CoMot strains can be regulated in a 3OC6HSL gradient generated by a second population of cells, we engineered non-motile *E*. *coli* sender strains that constitutively express a 3OC6HSL synthase (EsaI). Sender strains that produce different amounts of 3OC6HSL were designed by modifying the strength of the ribosome-binding site (RBS) upstream of *esaI*. We used two luminescent *E*. *coli* reporter strains with different dynamic ranges to quantify 3OC6HSL production by each sender strain. We observed that the sender strain with a weak RBS (weak-sender) produced approximately 10 µM 3OC6HSL and strain with the strong RBS (strong-sender) produced approximately 100 µM (Supplementary Fig. S5).

**Figure 7:**
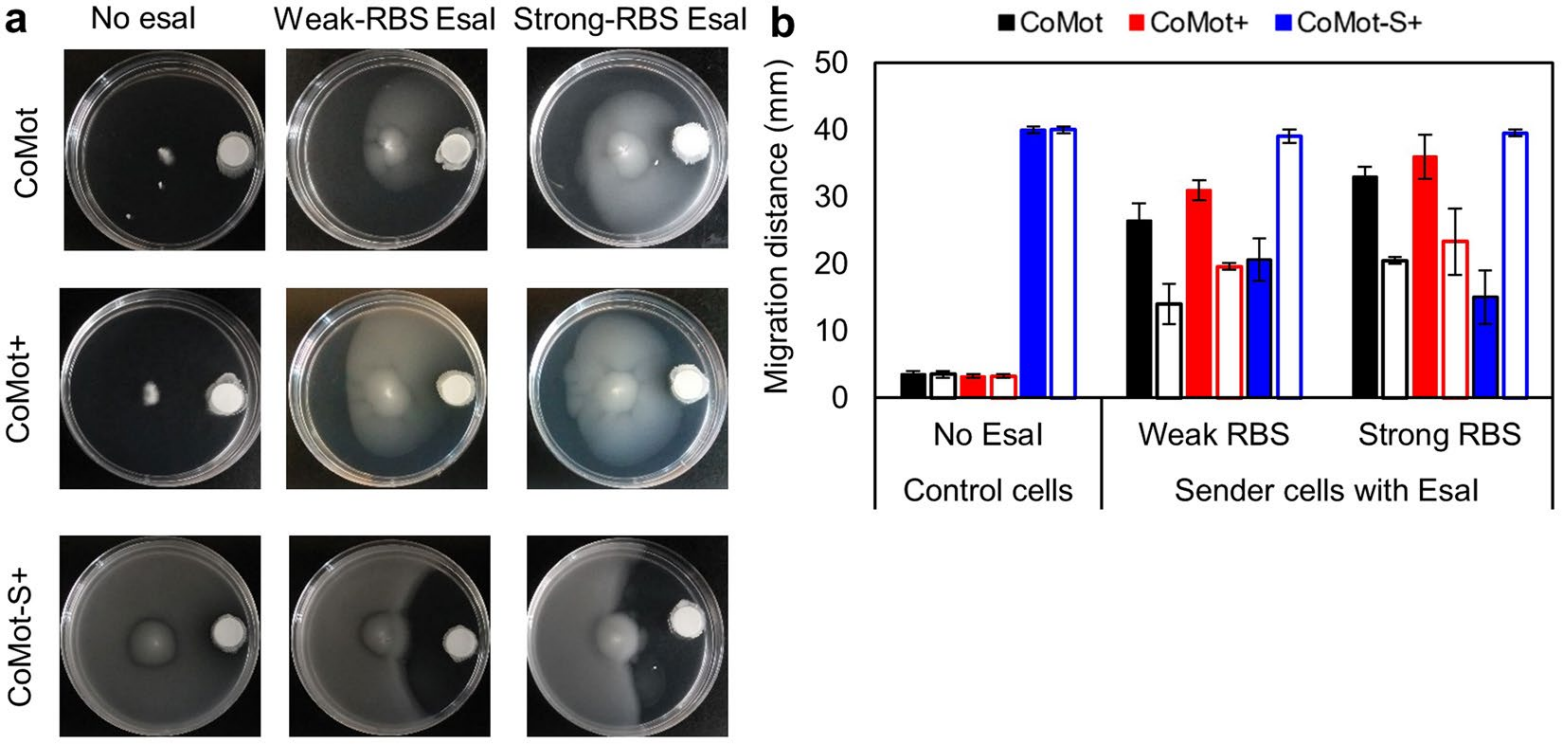
CoMot, CoMot+ and CoMot-S+ display directional movement in 3OC6HSL gradients generated by sender strains: (**a**) Control cells with no plasmid for EsaI expression, or sender cells where EsaI expression is controlled by a weak or strong RBS were used. Control, weak-sender and strong-sender cells were added on a Whatmann membrane and the plates were incubated for 8 h at 30 °C. CoMot, CoMot+ or CoMot-S+ were then inoculated at the centre of the plate and incubated at 30 °C for 36 h. Representative plate images are shown. (**b**) Plot of forward (solid bars) and reverse (open bars) migration distances for each sender/receiver combination. Error bars represent one standard deviation from the mean forward migration distance of three biological replicates.

We used motility assays to assess if the different senders are able to induce motility in the CoMot and CoMot-S strains (receivers). Sender strains were added on a membrane, placed on the plate surface, and incubated at 30 °C for 8 h prior to inoculation of a CoMot variant. As shown in Fig. 7a, the forward migration distance of CoMot cells inoculated onto plates with control cells that do not produce EsaI was comparable to levels of background migration observed in the absence of 3OC6HSL. CoMot cells inoculated onto plates with either sender strain were observed to be motile, and the forward migration distance with the strong-sender cells was significantly higher (*p* = 0.0028) than with the weak-sender cells. As expected, the more-sensitive CoMot+ cells showed a greater forward migration distance in response to both senders than the CoMot cells (Fig. 7b). No significant difference between the forward migration distances of CoMot+ cells was observed in response to the two sender cell strains. However, a higher density of CoMot+ cells was observed in response to the strong senders, indicating that motility was likely turned on in a larger population of CoMot+ cells (Fig. 7a). With the CoMot-S+ strain, migration to the edge of the plate was observed with the control cells. A decrease in forward migration distance was observed in the presence of both senders, where the decrease in forward migration distance was significantly higher in the presence of the strong-sender cells. With the weak senders, CoMot cells displayed a 1.9-fold (*p* = 0.0081) and CoMot+ cells a 1.6-fold (*p* = 0.00086) higher forward migration distance than reverse migration distance (distance between the inoculation point and the visible edge of cells that have migrated away from the senders). Similarly, CoMot and CoMot+ cells displayed 1.6-fold (*p* = 0.0025) and 1.5-fold (*p* = 0.035) higher forward than reverse migration distance with the strong senders. On the other hand, CoMot-S+ cells displayed a 1.9-fold (*p* = 0.015) lower forward migration distance than reverse migration distance in response to the weak senders and 2.6-fold lower (*p* = 0.015) with the strong senders. These observations indicate directional movement of CoMot and CoMot+ cells towards the senders and movement of CoMot-S+ away from the senders. The 3OC6HSL-insensitive strains (Δ*motA* transformed with plasmids containing P_*esaR*_-*motA* or P_*esaS*_-*motA*) did not display directional movement (Supplementary Fig. S6). Further, the modularity of the engineered sender-receiver architecture enables tuning of the 3OC6HSL gradient, motility response and the overall direction in which the population of cells migrates.

## Discussion

We have engineered QS signal-dependent motility in *E*. *coli* via transcriptional control of the motor protein, *motA*. In the absence of a gradient, the migration of cells was positively affected by the 3OC6HSL concentration and directional movement towards the signal source was observed in the presence of a signal gradient. Simulations of the migration response of CoMot cells further indicate that a gradient is essential for directional behaviour. The enhanced migration exhibited by cells in a signal gradient is remarkably similar to the behaviour of enzyme-based systems in their substrate gradients. For example, the increase in diffusivity of urease with increasing urea concentrations enables directed-propulsion of urease-coated beads up a gradient^27–29^. In our system, the motility of CoMot cells increases with increasing 3OC6HSL concentrations leading to enhanced diffusivity of cells that migrate to locations with signal concentrations sufficient to induce *motA* expression. This enhanced diffusivity enables population-level movement of cells up a signal gradient.

We also engineered CoMot-S cells that move down a 3OC6HSL gradient by changing the promoter controlling *motA* expression from the P_esaR_ to P_esaS_. Such a system, where cells can be manipulated to move up or down a signal gradient by switching one regulatory element, would be difficult to engineer with chemotaxis-based control, where a signal typically behaves as either an attractant or repellant^45, 46^. Although chemotaxis was not manipulated in CoMot cells, it is possible that chemotaxis enhances their directional behaviour in our experimental setup. At the beginning of the motility assays, any cell leaving the point of inoculation will move to a region of the plate that is nutrient rich. However, as the population distributes itself up and down the 3OC6HSL gradient, the CoMot cells that move up the 3OC6HSL gradient will continue to enter nutrient-rich regions, while those migrating down the gradient may enter regions potentially depleted of nutrients by the accumulated static cells. Therefore, chemotaxis may enhance migration towards nutrient-rich regions that are available up the gradient and increase the forward migration distance.

While chemotaxis-enabled directional movement of cells occurs at the individual-cell level^15^, the directional movement of CoMot cells occurs at the population-level. Further studies are required to explore if CoMot cells will exhibit observable levels of enhanced-diffusivity up a 3OC6HSL gradient at the microscopic-level and whether this type of control can be used for applications in microfluidic devices. The time required for non-uniform distribution of CoMot cells in signal gradient, which requires transcription, translation and cell movement, is also expected to be much longer than a chemotaxis-based system, which requires only protein phosphorylation^47, 48^. These two time-scales may be advantageous for different applications, and could potentially be combined for precise cellular movement in response to signal molecules. Previously, a population of *Serratia* cells was attached to a 10 μm-sized piece of polymer to generate a micro-bio-robot. The motility of the cells was used to propel the robot. However, the direction of movement was controlled using external magnetic fields^49^. An understanding of the behaviour of CoMot cells under microfluidic conditions could enable their use as actuators of both motion and direction for micro-bio-robots or targeted-delivery systems.

Pairing our QS-responsive receiver strains, CoMot, CoMot+ and CoMot-S+, with 3OC6HSL-producing sender strains demonstrated that our motility-control system is both modular and tuneable. As described above, the use of EsaR variants and *esa* promoter variants enabled the engineering of receiver strains that display a range of behaviours in terms of signal sensitivity, whether motility is turned on or off in the presence of 3OC6HSL, and the directionality of movement in a signal gradient. The amount of 3OC6HSL produced by the sender cells was tuned by varying the strength of the RBS upstream of *esaI*. The ability to manipulate motility in CoMot cells using sender cells could enable the use of our sender-receiver system to detect other signals of interest. Detection of target signals using the sender-receiver system can be achieved by swapping the constitutive promoter controlling *esaI* expression to one regulated in response to any target signal of interest. Advantages of this architecture for biosensing-system design include modularity and signal amplification via QS^50^. Further, logic gate-type behaviours in the receivers may enable more complex sensing systems^51, 52^. For example, multiple senders engineered to detect different target molecules and produce 3OC6HSL could be combined to generate a modular, tuneable OR-type sensor system that is turned on by any one of the target molecules. Multiple intermediary QS signals could be used to generate a broader range of signal integration circuits. These systems can be applied for biosensing and bioactuation in complex environments, such as a tumour, soil or the human gastrointestinal tract, where recognition, integration and reporting of multiple signal inputs is advantageous.

We have demonstrated that transcriptional regulation of a motility gene allows for control of cell motility and enables directional movement in gradients generated by exogenously added signal or *in-situ* bio-production. The strategy used here presents a robust mechanism for controlling the directional movement of a population of cells without the manipulation of chemotaxis. We anticipate that these engineered cells will find application as actuators for micro-bio-robots and as drivers of motility-dependent pattern formation. The modular sender-receiver architecture, combined with the population-level control of directional movement, sets the stage for development of biosensing frameworks where senders are engineered to detect target stimuli and produce a QS molecule, and cellular motility in the receivers serves as novel biosensing output.

## Methods

### Plasmid Construction and strains

The *motA*-deletion strain (∆*motA*), *E*. *coli* RP6666^39^, was used in this study. Plasmids and primers used in this study are listed in Supplementary Tables S2 and S3. Sequences of the promoters and ribosome binding sites (RBS) used in this study are provided in Supplementary Table S4 and S5. To engineer the receiver strains, we constructed a two-plasmid system consisting of a *motA-*expression plasmid and an *esaR*-expression plasmid. pCS-P_*esaR*_ *-motA-gfp* and pCS-P_*esaS*_ *-motA-gfp* were used for expression of *motA*. To construct pCS-P_*esaR*_ *-motA-gfp*, the P_*esaR*_ promoter, *motA* and *gfp* were cloned between the *Xho*I and *Not*I sites of a low-copy, pCS26 plasmid. We tuned the strength of the RBS upstream of *motA* and *gfp* using the RBS calculator^53, 54^ and the 5′ primer was used to add the modified RBSs to the genes. *motA* was PCR-amplified from pDFB36^41^ using the primers 5′-SMotA-*Kpn*I and 3′-MotA-*BamH*I. P_*esaR*_ was PCR-amplified from pCS-P_*esaR*_-*gfp* using the primers 5′-P_*esaR*_-*Xho*I and 3′-P_*esaR*_-*Kpn*I-SMotA. The amplified P_*esaR*_ and *motA* were assembled using assembly PCR with the primers 5′-P_*esaR*_-*Xho*I and 3′-MotA-*BamH*I. *gfp* was PCR-amplified from pCS- P_σ70_-*gfp* using the primers 5′-*Not*I-SGFP and 3′-*Bgl*II. To construct pCS-P_*esaS*_ *-motA-gfp*, the P_*esaS*_ promoter was PCR-amplified from pCS-P_*esaS*_-*lux* ^55^ using the primers ZEO5 and 3′-*Kpn*I*-*P_*esaS*_. The amplified product was digested with *Xho*I and *Kpn*I and ligated into *Xho*I and *Kpn*I-digested pCS-P_*esaR*_ *-motA-gfp*. pAC- P_σ70_-*esaR* and pAC- P_σ70_-*esaR-D91G* ^43^ were used as *esaR*-expression plasmids. In these plasmids, a P_σ70_-dependent promoter and either *esaR* or *esaR-D91G* genes were cloned between the *Xba*I and *BamH*I sites of the medium-copy, pACYC184 plasmid. To construct the CoMot and CoMot+ strains, ∆*motA* competent cells were transformed with pCS-P_*esaR*_ *-motA-gfp* and pAC- P_σ70_-*esaR* or pAC- P_σ70_-*esaR-D91G* respectively. Similarly, to construct the CoMot-S and CoMot-S+ strains, pCS-P_*esaS*_ *-motA-gfp* and pAC- P_σ70_-*esaR* or pAC- P_σ70_-*esaR-D91G* were transformed into ∆*motA* cells.

For construction of the sender strains, two *esaI* expression plasmids, pAC-P_*lac*_-(RBS_weak_)*esaI* or pAC-P_*lac*_-(RBS_strong_)*esaI*, were constructed. To construct pAC-P_*lac*_-(RBS_strong_)*esaI*, *esaI* with the strong-RBS was amplified from pAC-P_*lac*_-*esaR-esaI* ^55^ using the primers 5′-*Kpn*I-*esaI* and 3′-*BamH*I-*esaI*. The amplified product was digested with *Kpn*I and *BamH*I and ligated into *Kpn*I and *BamH*I-digested pAC-P_*lac*_-*esaR* ^43^. To construct pAC-P_*lac*_-(RBS_weak_)*esaI*, the P_*lac*_ promoter was amplified from pAC-P_*lac*_-*esaR-esaI* ^55^ using the primers 5′-pAC-promseq and 3′-P_*lac*_-*BamH*I. The amplified product was digested with *Xba*I and *BamH*I and ligated into *Xba*I and *BamH*I-digested pAC-P_σ70_-*esaR-esaI* ^56^. For construction of the sender strains, ∆*motA* competent cells were transformed with pCS26 and an *esaI* expression plasmids. For construction of control strains, ∆*motA* competent cells were transformed with pCS26 and pACYC184. *E*. *coli* DH5α strain was used in all cloning procedures.

### Overnight cultures

Overnight cultures were made by inoculating single colonies of strains picked from Luria Broth (LB) agar plates in 5 mL of LB media with chloramphenicol (50 μg/mL) and kanamycin (50 μg/mL). The cultures were incubated overnight at 37 °C with shaking (225 rpm).

### Motility assays

Semi-solid media consisted of 1% tryptone, 0.5% NaCl and 0.25% agar with chloramphenicol (25 μg/mL) and kanamycin (25 μg/mL). For assays requiring a uniform 3OC6HSL concentration across the plate, 3OC6HSL was directly added to the media before pouring into petri dishes. To establish a 3OC6HSL gradient, 0.002 to 0.2 µmoles of 3OC6HSL was added to a Whatmann membrane (Grade 3–6 µm, diameter: 1.2 cm). 10 μM of 3OC6HSL would be the final concentration if 0.2 µmoles of 3OC6HSL diffused uniformly through the plate. The centre of the membrane was placed 1.25 cm from the edge of the plate and 3OC6HSL was allowed to diffuse from the membrane into the media at room temperature for 8 h prior to inoculation unless otherwise indicated. In the sender-receiver motility assays, overnight cultures of the sender strains were concentrated 10-fold by centrifuging the cultures and re-suspending them in LB. 10 μL of the concentrated cultures were added to a Whatmann membrane, which was placed 1.25 cm from the edge of the plate and incubated at 30 °C for 8 h prior to inoculation with the receiver cells. For all motility assays, receiver cells were inoculated using a pipette tip containing 1 μL of overnight culture. The tip was inserted at the centre of the plate, approximately 3 mm below the surface of the media, and the cells were ejected as the tip was pulled up through the media. To assess migration, images were obtained up to 48 h after incubation at 30 °C.

### Quantitative characterization of 3OC6HSL produced by sender strains

Overnight cultures of sender strains were diluted 100-fold in 5 mL LB with appropriate antibiotics and grown at 37 °C with shaking (225 rpm) for 8 h. 1 mL of the culture was centrifuged at 16,000 rcf for 3 minutes. The supernatant was filter sterilized using a 0.2 μM polyether sulfone filer. Two *E*. *coli* reporter strains (DH5α cells transformed with (i) pCS-P_*esaR*_-*lux* and pAC-P_σ70_-*esaR-I70V*/*D91G* (ii) pCS-P_*esaR*_-*lux* and pAC- P_σ70_-*esaR* ^43^) that luminesces in a 3OC6HSL concentration-dependent manner were used for quantification of 3OC6HSL produced by the sender cells, by comparing luminescence in response to 3OC6HSL in the supernatants to the response to known amounts of 3OC6HSL. The quantitative characterization of 3OC6HSL using the reporter strain was performed as described by *Shong et al*.^43^.

### Statistical analysis

Two-tailed paired t-tests were applied to evaluate significance when required. *p* values are reported in the text for each statistical test.

### Modelling of signal molecule-guided bacterial motility

All simulations were run in COMSOL Multiphysics version 5.1. A 2-dimensional version of the experimental set up was simulated in the model. Circular, 8 cm-diameter plates were used in all simulations except in pattern-formation simulations. In pattern-formation simulations, either circular 15 cm-diameter plates or 13 × 13 cm square plates were used. Neumann boundary conditions are imposed on Equations (1) and (3) to ensure that motile cells and the signal molecule do not diffuse beyond the boundaries. All simulations were started with 3.5*10^7^ static cells/m^2^ (equivalent of 1000 cells) as the inoculum and 170 μmoles/m^2^ of the signal as the signal source and run for a simulation time of 24 h unless otherwise noted. The location of the cells (inoculation point) and signal source were similar to the experimental set up. When reported, forward and reverse migration distances were measured as distances from the inoculation point towards and away from the signal source where a total cell concentration (m + s) ≥ 10^8^ cells/m^2^ was observed. In all simulated plate images log (total cell concentration) is shown. To simulate the migration response of CoMot and CoMot+ cells *K*_2_ values of 2.5 and 1 nmoles/cm^2^ were used respectively. In the studies where simulations were run varying the value of each parameter, the gradient was established using 85 μmoles/m^2^ of the signal near edge of the plate (migration distance = 4 cm).

## Electronic supplementary material

Supplementary information
