# Supplementary material for: Transcriptional control of motility enables directional movement of Escherichia coli in a signal gradient
Source: Sci Rep. 2017 Aug 21;7:8959. doi: 10.1038/s41598-017-08870-6 (PMC5566481; doi:10.1038/s41598-017-08870-6)
Supplement: Supplementary file 1 — Supplementary information [file 41598_2017_8870_MOESM1_ESM.pdf]

Transcriptional control of motility enables directional movement of Escherichia coli in a signal gradient

Jayamary Divya Ravichandar†‡, Adam G. Bower†‡[#], A. Agung Julius₴ and Cynthia H. Collins†‡§*

† Department of Chemical and Biological Engineering, Rensselaer Polytechnic Institute, 110 8th Street, Troy, New York 12180, United States of America

‡ Center for Biotechnology and Interdisciplinary Studies, Rensselaer Polytechnic Institute, 110 8th Street, Troy New York 12180, United States of America

[#] Regeneron Pharmaceuticals, Rensselaer, New York 12144, United States of America

₴ Department of Electrical, Computer and Systems Engineering, Rensselaer Polytechnic Institute, 110 8th Street, Troy, New York-12180, United States of America

§ Department of Biological Sciences, Rensselaer Polytechnic Institute, 110 8th Street, Troy, New York-12180, United States of America

* Phone: 518-276-4178 Fax: 518-276-4233. E-mail: ccollins@rpi.edu.

Supplementary information:

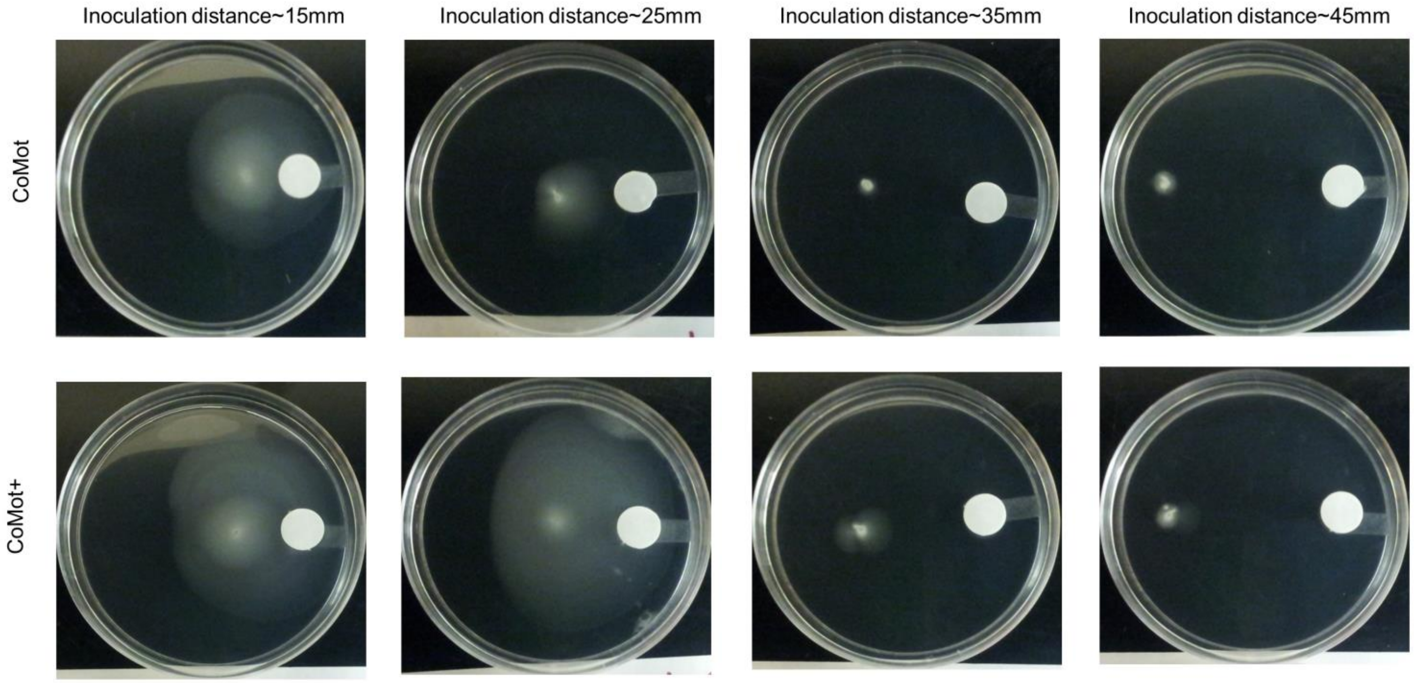

Supplementary figure S1: The inoculation distance of CoMot and CoMot+ cells from the 3OC6HSL source affects their migration response: To establish a 3OC6HSL gradient, 5.3µg of 3OC6HSL was added on a membrane and it was allowed to diffuse into the media for 8h prior to inoculation of CoMot and CoMot+ cells. Cells were inoculated at increasing distances from the source to quantify the effect of inoculation distance on migration response. The assay was set up in triplicate for each strain and representative images of plates after 24 h of incubation at 30°C are shown.

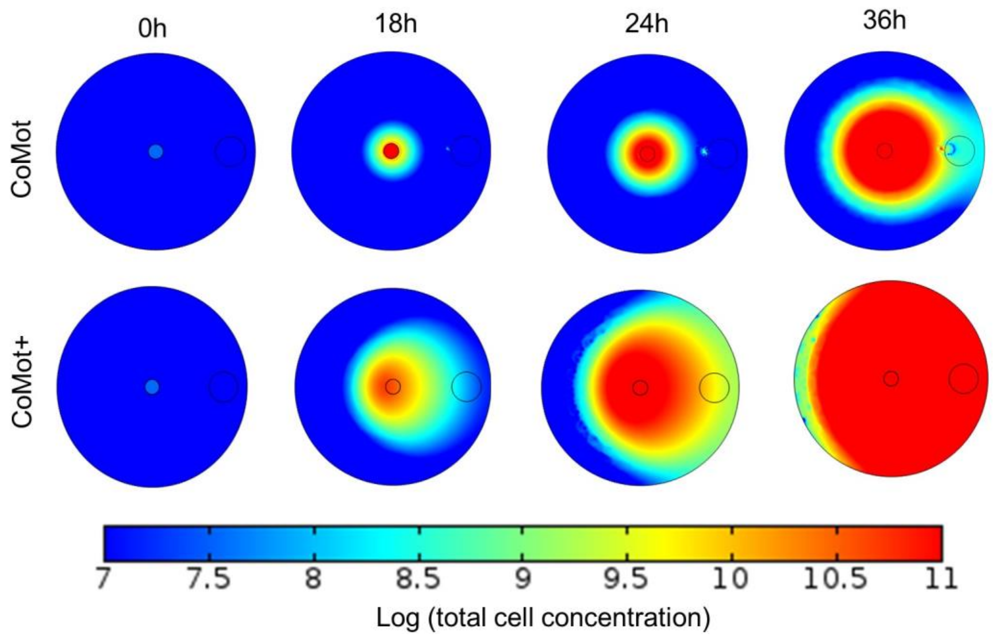

Supplementary figure S2: Time course simulation of CoMot and CoMot+ cells in a signal gradient shows directional movement towards the signal source: Signal gradients were simulated by using an initial signal concentration of 170 µmoles/m^2 on a membrane that was membrane was placed 1.25 cm from the edge of the plate. 3.5*10^7 CoMot or CoMot+ cells/m^2 was used as the inoculum at the centre of the plate. The log (total cell concentration) indicated in the images were obtained following simulation times of 0, 18, 24 and 36 h. To simulate the migration response of CoMot and CoMot+ cells K_2 values of 100 and 1 nmoles/cm^2 were used, respectively.

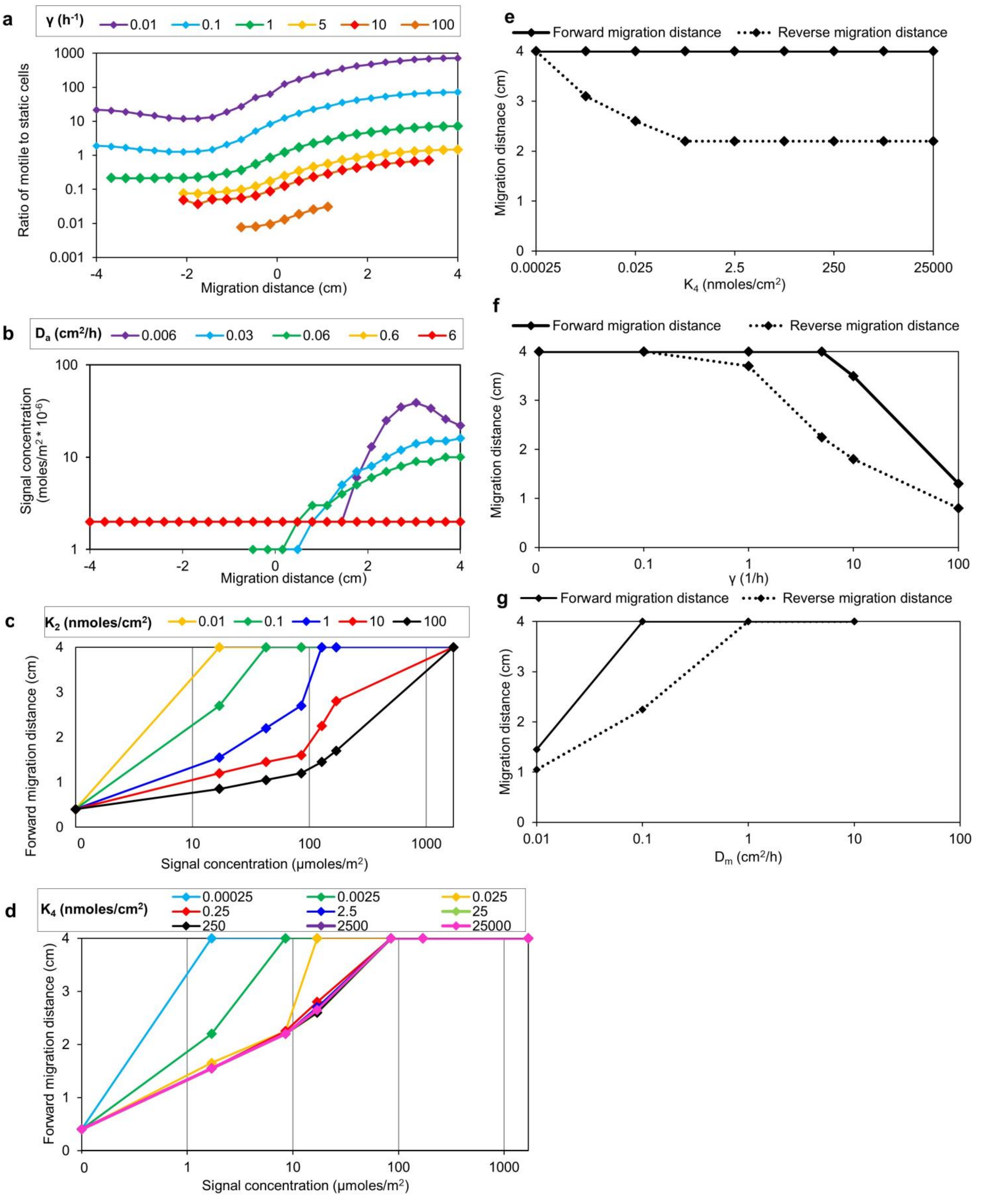
a
γ (h-1)
0.01
0.1
1
5
10
100
Ratio of motile to static cells
1000
100
10
1
0.1
0.01
0.001
-4
-2
0
2
4
Migration distance (cm)
b
Da (cm2/h)
0.006
0.03
0.06
0.6
6
Signal concentration (moles/m2 * 10-6)
100
10
1
Migration distance (cm)
c
K2 (nmoles/cm2)
0.01
0.1
1
10
100
Forward migration distance (cm)
Signal concentration (μmoles/m2)
0
10
100
1000
d
K4 (nmoles/cm2)
0.00025
0.0025
0.025
0.25
2.5
25
250
2500
25000
Forward migration distance (cm)
Signal concentration (μmoles/m2)
0
1
10
100
1000
e
Forward migration distance
Reverse migration distance
Migration distance (cm)
0.00025
0.025
2.5
250
25000
K4 (nmoles/cm2)
f
Forward migration distance
Reverse migration distance
Migration distance (cm)
0
0.1
1
10
100
γ (1/h)
g
Forward migration distance
Reverse migration distance
Migration distance (cm)
0.01
0.1
1
10
100
Dm (cm2/h)

Supplementary figure S3: Effect of the rate of switching from motile to static (*γ*), diffusivity of the signal (D_a), sensitivity of cells to the signal (K_2 and K_4) and effective diffusivity of cells (D_m) on the system: In the simulations, signal gradients were simulated by using an initial signal concentration of 85 μmoles/m^2 on a membrane that was membrane was placed 1.25 cm from the edge of the plate. 3.5*10^7 static cells/m^2 was used as the inoculum at the centre of the plate (migration distance = 0 cm). Results after a simulation time of 24 h are shown. **(a)** γ was varied from 0.01 – 100h^{-1} and all other parameters were held constant at values defined in the base parameter set. For each value of γ, the ratio of motile to static cells (m/s) across the diameter of the plate (migration distance = -4 to 4 cm) was plotted. The ratio was only calculated at points with a total cell concentration ≥10^8 cells/m^2. **(b)** Simulations were run by varying D_a from 0.01 - 10cm^2/h while holding all other parameters constant. For each value of D_a, the signal concentration across the diameter of the plate (migration distance = -4 to 4 cm) was plotted. **(c)** The response of cells was simulated using K_2 values in the 0.01 – 100 nmoles/cm^2 range. For each K_2, the response of cells to gradients established using signal concentrations in the 0 – 1700 nmoles/m^2 range was simulated and the forward migration distance was measured as distances from the inoculation point (migration distance = 0) towards the signal source at which a total cell concentration ≥10^8 cells/m^2 was observed. **(d)** Similarly, K_4 was varied from 0.00025 to 25000 nmoles/cm^2. The response of cells to gradients established using signal concentrations in the 0 – 1700 nmoles/m^2 range was simulated and the forward migration distance was determined. **(e)** For each simulated K_4, the forward and reverse migration distances in response to 85 μmoles/m^2 of the signal are plotted. Forward and reverse migration distances were measured as distances from the inoculation point (migration distance = 0) towards and away from the signal source at which a total cell concentration ≥10^8 cells/m^2 was observed. **(f)** Simulations were run varying γ from 0 – 100h^{-1}. Forward and reverse migration distances were measured for each simulated γ. **(g)** Simulations were run by varying D_m from 0.01-10cm^2/h. Forward and reverse migration distances were measured for each simulated D_m.

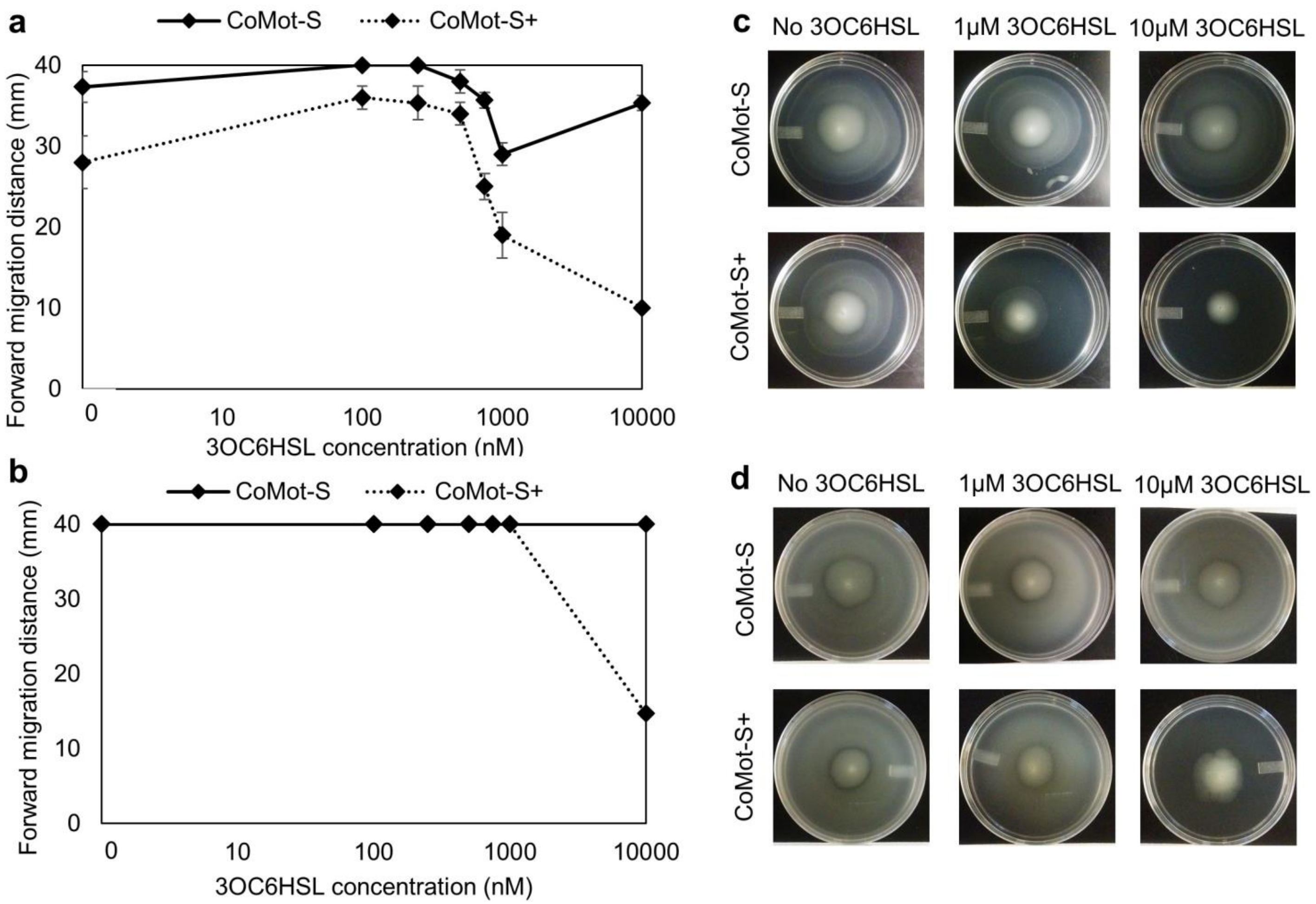

Supplementary figure S4: CoMot-S and CoMot-S+ cells show 3OC6HSL-dependent decrease in migration on plates with different uniform 3OC6HSL concentrations: (a) CoMot-S and CoMot-S+ cells were inoculated on plates with 3OC6HSL concentrations ranging from 0 - 10μM. Migration radius was measured as the distance between the inoculation point and the visible edge of migration of cells on the plate after 24 h of incubation at 30°C. Error bars represent one standard deviation from the mean migration radius of three biological replicates. **(b)** Migration radius measured after 36 h of incubation at 30°C **(c)** Representative plate images from the assay after incubation for 24 h and **(d)** 36 h.

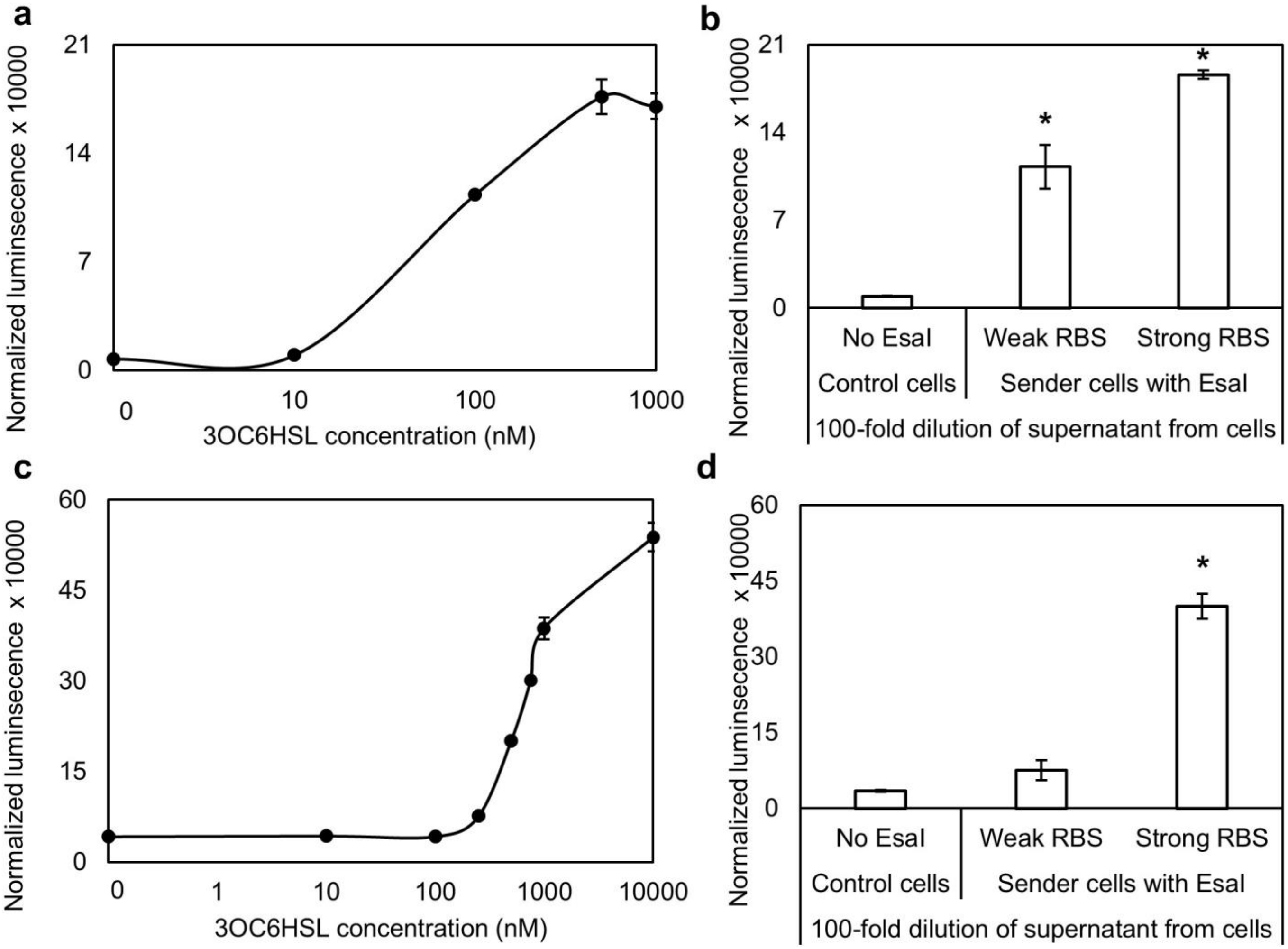

Supplementary figure S5: Quantitative assessment of 3OC6HSL produced by sender strains: **(a)** 3OC6HSHL-dependent luminescence response of *E. coli* reporter cells transformed with plasmids containing P_{esaR}-*lux* and $P_{\sigma 70}$-*esaR170V/D91G* **(b)** Luminesce of this reporter in response to a 100-fold dilution of supernatants collected from sender-cell cultures **(c)** 3OC6HSHL-dependent luminescence response of *E. coli* reporter cells transformed with plasmids containing P_{esaR}-*lux* and $P_{\sigma 70}$-*esaR* **(d)** Luminesce of this reporter in response to a 100-fold dilution of supernatants collected from sender-cell cultures Error bars represent one standard deviation from the mean luminescence of three biological replicates. Paired t-tests were used to evaluate significance (p<0.02). Asterisk indicates luminesce that were significantly higher than the luminesce observed in response to 3OC6HSL in the supernatants from no-EsaI control cells.

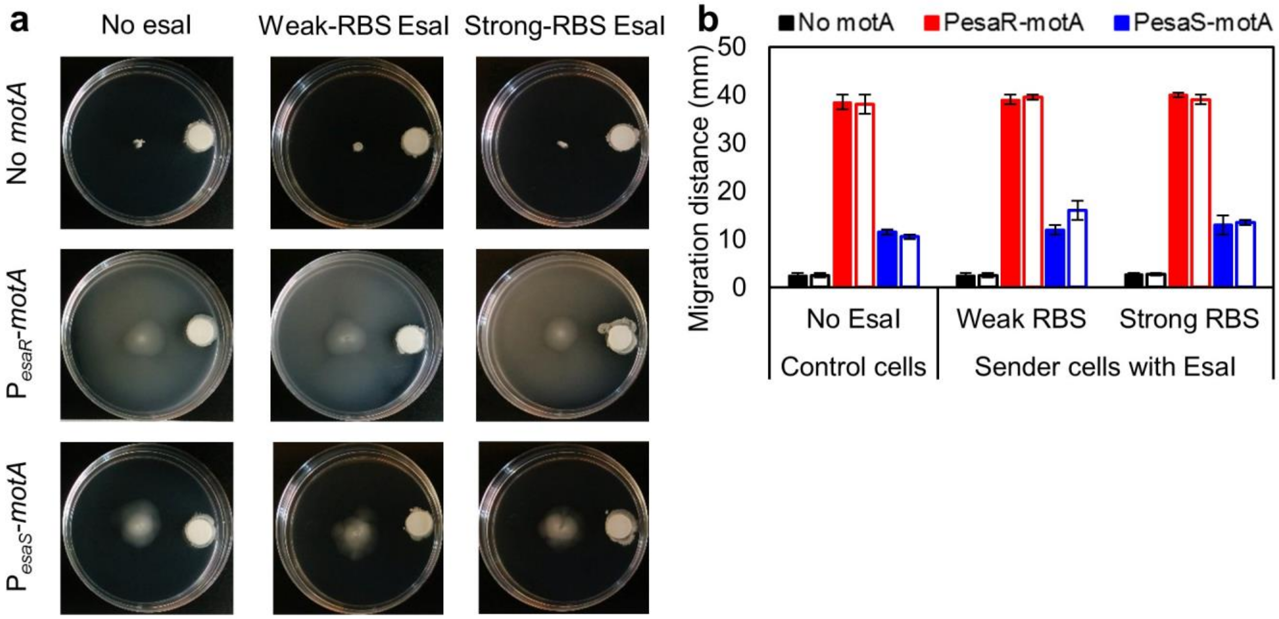

Supplementary figure S6: 3OC6HSL-insensitive strains lacking EsaR do not display directional movement in 3OC6HSL gradients generated by sender strains: (a) Control cells with no plasmid for EsaI expression, or sender cells where EsaI expression is controlled by a weak or strong RBS were used. Control, weak-sender and strong-sender cells were added on a Whatmann membrane and the plates were incubated for 8 h at 30°C. Δ*motA* transformed with plasmids expressing no *motA*, P_{esaR}-*motA* or P_{esaS}-*motA* were then inoculated at the centre of the plate and incubated at 30°C for 36 h. Representative plate images are shown. **(b)** Plot of forward (solid bars) and reverse (open bars) migration distances for each sender/control combination. Error bars represent one standard deviation from the mean forward migration distance of three biological replicates.

Supplementary table S1: Values used in the base parameter set

Parameter	Value	Choice of the value used
Growth rate (λ)	0.5 h^{-1}	From literature[1,2]
Rate of switching from static to motile (k_1)	10 h^{-1}	The key components captured by the parameter k_1 are the rates of synthesis of MotA and MotA-dependent restoration of motility in cells. We estimated a MotA synthesis rate of 30 – 90 h^{-1} based on the typical rates for gene transcription[3] and protein translation[4] in *E. coli*. Since four MotA proteins are required per stator of the *E. coli* flagellar motor[5] and motility in restored in a stepwise manner with the addition of each stator[6], a base value of 10 $h^{-1}$was used for k_1.
Rate of switching from motile to static (γ)	5 h^{-1}	Based on typical protein decay rates in *E. coli*[7]
Sensitivity of cells to *A* when switching from static to motile (K_2)	100 nmoles/cm^2 (CoMot)<br>1 nmoles/cm^2 (CoMot+)	Simulations were run varying K_2 to identify values that captured experimentally observed sensitivity of CoMot+ cells to the signal. A value of 1 nmoles/ cm^2 approximately captured the response of CoMot+ cells. *Shong et al.* have demonstrated a 100-fold difference in the half maximal 3OC6HSL-sensitivity of *E. coli* luminescent reporter strains using the wild-type or EsaR-D91G repressor[8]. Based on this, we simulated the difference in response of CoMot and CoMot+ to 3OC6HSL using K2 values of 100 and 1 nmoles/cm^2.
Sensitivity of cells to *A* when switching from static to motile (K_4)	2.5 nmoles/cm^2	A K_4 in the same range of that utilized for K_2 was used in initial simulations. Since parametric studies revealed that K_4 only had a small effect on system behavior, it was not varied in simulation for CoMot & CoMot+.
Effective diffusivity of cells (D_m)	0.1 cm^2/h	D_m was measured experimentally by inoculating CoMot+ cells on a plate with 1 μM 3OC6HSL and measuring the migration diameter over a time course of 24 h. D_m was estimated as 0.1 cm^2/h from this assay (average migration area/time). The experimentally-estimated D_m accounts for drag and mechanical forces on cells from the agar in the plate.
Diffusivity of the signal molecule (D_a)	0.06 cm^2/h	From literature [9]

Supplementary table S2: Plasmids used in this study

Plasmid	Description	Reference
**pDFB36**	Plasmid with *motA* downstream of a lactose inducible promoter	Blair *et al.* [6]
**pCS26**	Low-copy plasmid backbone	Dr. M.Surette
**pCS-P_{esaR}-*gfp***	Plasmid with *gfp* downstream of P_{esaR}	Shong *et al.*[10]
**pCS- $P_{\sigma70}$-*gfp***	Plasmid with *gfp* downstream of a σ^{70}-dependent promoter	Dr. M.Surette
**pCS-P_{esaR}-*motA-gfp***	*motA*-expression plasmid with *motA* and *gfp* downstream of P_{esaR}	This study
**pCS-P_{esaS}-*motA-gfp***	*motA*-expression plasmid with *motA* and *gfp* downstream of P_{esaS}	This study
**pAC- $P_{\sigma70}$-*esaR***	*esaR*-expression plasmid with *esaR* downstream of a σ^{70}-dependent promoter	Shong *et al.* [11]
**pAC- $P_{\sigma70}$-*esaRD91G***	*esaR*-expression plasmid with *esaR-D91G* downstream of a σ^{70}-dependent promoter	Shong *et al.* [11]
**pACYC184**	Medium-copy plasmid backbone	NEB
**pAC-P_{lac}-(RBS_{strong})*esaI***	*esaI*-expression plasmid with *esaI* with a strong RBS downstream of a P_{lac} promoter	This study
**pAC-P_{lac}-(RBS_{weak})*esaI***	*esaI*-expression plasmid with *esaI* with a weak RBS downstream of a P_{lac} promoter	This study
**pAC-P_{lac}-*esaR-esaI***	Plasmid with *esaR* and *esaI* downstream of a P_{lac} promoter	Shong *et al.*[12]
**pAC-P_{lac}-*esaR***	Plasmid with *esaR* downstream of a P_{lac} promoter	Shong *et al.* [11]
**pAC- $P_{\sigma70}$-*esaR-esaI***	Plasmid with *esaR* and *esaI* downstream of a σ^{70}-dependent promoter	Shong *et al.*[10]
**pCS-P_{esaR}-*lux***	Plasmid with *lux* (luminescence gene) downstream of P_{esaR}	Shong *et al.* [11]
**pAC- $P_{\sigma70}$-*esaR-I70V/D91G***	Plasmid with *esaR-I70V/D91G* downstream of a σ^{70}-dependent promoter	Shong *et al.* [11]

Supplementary table S3: Primers used in this study

Primer	Nucleotide sequence
**5'-SMotA-*Kpn*I**	ccgcggaagggaacttccgtttataaggttagaatgcttatcttattaggttacctggt
**3'-MotA-*BamH*I**	ctagtcggatccttatcatgcttcctcggttgtcgtctg
**5'-P_{esaR} -*Xho*I**	ctgcaactcgaggcagattgagtaaccgtgaatgtttg
**3'-P_{esaR}-*Kpn*I-SMotA**	taaacggaagaacccttccgcggggtaccgctgcttcttttacttaacgtggac
**5'-*Not*I-SGFP**	ctgcaagcggccgcggcgattaatcaacataaaattaaggaggtaaggaatgcgtaaaggagaggaacttttca
**3'-*Bgl*II**	ctgatcagatctcggatttgtcctactcaggagagc
**ZEO5**	ccagctggcaattccga
**3'-*KpnI*-P_{esaS}**	tggctcggtaccaaacaactgaagccattgtaacctct
**5'-*Kpn*I-*esaI***	ttgacaggtaccatgctggagctgttcgacgttagc
**3'-*BamH*I-*esaI***	ttcagtggatccttattacaccggcagggtcagcg
**5'-pAC-promseq**	gattacgcgcagaccaaaacgatc
**3'-P_{lac}-*BamH*I**	tacagtggatcctgtttcctgtgtgaaattgttatccg

Supplementary table S4: Sequence of promoters used in this study

Promoter	Nucleotide sequence
**P_{esaR}**	gcagattgagtaaccgtgaatgtttgtacaaatgtttcaaagatgttactatgagtgtcccggccagcatcactttatattttgtgacgtctggccggacg ttttccctagtgttggctgttttagcgacctggccgtacaggtcaggtttttttttaccgctaaacaactgaagccattgtaacctctgaatgattcattgtaag ttactcttaagtatcatcttgcctgtactatagtgcaggttaagtccacgttaagtaaaagaagcagc
**P_{esaS}**	catttgaaggattttttttgctcacaacagtgtaagcgtaatccggactacccagcggagataactttctctgtatgtaagtctgaagcgtatccgttattgt ttgattttcaaggaaaaaagaaaacattcaggctccatgctgcttcttttacttaacgtggacttaacctgcactatagtacaggcaagatgatacttaa gagtaacttacaatgaatcattcagaggttacaatggcttcagttgttt
**$P_{\sigma70}$**	aataattctttacatttatgcttccggctcgtattctacgtgcaatt

**P_{lac} and *lac* operon**	ccaatacgcaaaccgcctctccccgcgcgttggccgattcattaatgcagctggcacgacaggtttcccgactggaaagcgggcagtgagcgcaacgcaattaatgtgagttagctcactcattaggcaccccaggctttacactttatgcttccggctcgtatgttgtgtggaattgtgagcggataacaatttcacacaggaaaca

Supplementary table S5: Sequence of ribosome binding sites (RBS) used in this study

RBS	Nucleotide sequence
**RBS upstream of *motA***	ccgcggaagggaacttccgtttataaggttaga
**RBS upstream of *gfp***	ggcgattaatcaacataaaattaaggaggtaagga
**RBS upstream of *esaR* and *esaR-D91G***	taaagaggagaaa
**Weak RBS upstream of *esaI***	cgcgagggccgcagtaacttttaagaggaaatgga
**Strong RBS upstream of *esaI***	taaagaggagaaa

References:

1. Balagaddé, F. K. *et al.* A synthetic Escherichia coli predator-prey ecosystem. *Mol. Syst. Biol.* **4,** 187; 10.1038/msb (2008).

2. Lendenmann, U., Snozzi, M. & Egli, T. Growth kinetics of Escherichia coli with galactose and several other sugars in carbon-limited chemostat culture. *Can. J. Microbiol.* **46,** 72–80 (2000).

3. Vogel, U. & Jensen, K. F. The RNA chain elongation rate in Escherichia coli depends on the growth rate. *J. Bacteriol.* **176,** 2807–2813 (1994).

4. Young, R. & Bremer, H. Polypeptide-chain-elongation rate in Escherichia coli as a function of growth rate. *Biochem. J.* **160,** 185–194 (1976).

5. Garza, A. G., Harris-Haller, L. W., Stoebner, R. A. & Manson, M. D. Motility protein interactions in the bacterial flagellar motor. *Proc. Natl. Acad. Sci. U. S. A.* **92,** 1970–1974 (1995).

6. Blair, D. F. & Berg, H. C. Restoration of torque in defective flagellar motors. *Science* **242,** 1678–1681 (1988).

7. Basu, S., Gerchman, Y., Collins, C. H., Arnold, F. H. & Weiss, R. A synthetic multicellular system for programmed pattern formation. *Nature* **434,** 1130–1134 (2005).

8. Shong, J., Huang, Y.-M., Bystroff, C. & Collins, C. H. Directed evolution of the quorum-sensing regulator EsaR for increased signal sensitivity. *ACS Chem. Biol.* **8,** 789–795 (2013).

9. Hense, B. a, Müller, J., Kuttler, C. & Hartmann, A. Spatial heterogeneity of autoinducer regulation systems. *Sensors (Basel).* **12,** 4156–4171 (2012).

10. Shong, J. Quorum-sensing repressor-based tools for cell-cell communication in synthetic biology. *PhD thesis*, *Rensselaer Polytechnic Institute, Department of Chemical and Biological Engineering*. (2013).

11. Shong, J., Huang, Y.-M., Bystroff, C. & Collins, C. H. Directed Evolution of the Quorum-Sensing Regulator EsaR for Increased Signal Sensitivity. *ACS Chem. Biol.* **8,** 789–795 (2013).

12. Shong, J. & Collins, C. H. Engineering the esaR promoter for tunable quorum sensing-dependent gene expression. *ACS Synth. Biol.* **2,** 568–575 (2013).
